# Association of Triglyceride-Glucose-Frailty Index with Cardiovascular Disease and All-Cause Mortality Incidence in Individuals with Cardiovascular-Kidney-Metabolic Syndrome Stages 0–3: A Nationwide Prospective Cohort Study

**DOI:** 10.3390/jcm15114156

**Published:** 2026-05-28

**Authors:** Xingsheng Ye, Yuanqi Chen, Wenjie Peng, Miaomiao Yang, Daoliang Zhang

**Affiliations:** 1 Department of Cardiology, Fuwai Shenzhen Hospital, Chinese Academy of Medical Sciences, Shenzhen 518057, China; 2 Department of Internal Medicine-Cardiovascular, Guangzhou Twelfth People’s Hospital, Guangzhou 510620, China; 3 Department of Special Care Center, Fuwai Hospital, National Clinical Research Center for Cardiovascular Diseases, National Center for Cardiovascular Diseases, Chinese Academy of Medical Sciences and Peking Union Medical College, Beijing 100037, China

**Keywords:** triglyceride-glucose index, frailty index, cardiovascular disease, all-cause mortality, cardiovascular-kidney-metabolic syndrome

## Abstract

**Background**: Both the triglyceride-glucose (TyG) index and frailty are associated with cardiovascular disease (CVD) incidence and all-cause mortality, yet the combined impact of the TyG-Frailty Index (TyGFI) remains insufficiently explored, particularly in individuals with cardiovascular-kidney-metabolic (CKM) syndrome stages 0–3. **Methods**: This study included middle-aged and elderly adults from the China Health and Retirement Longitudinal Study (CHARLS). The outcomes were the incidence of CVD and all-cause mortality. Multivariable Cox proportional hazards models, Kaplan–Meier curves, and restricted cubic spline (RCS) analyses were used to assess the association between TyGFI and the incidence of CVD and mortality. The predictive performance of different indices was compared using receiver operating characteristic (ROC) curves. Subgroup analyses were employed to assess the influence of different sociodemographic and clinical characteristics. Mediation analysis was conducted to evaluate whether age mediates the association between TyGFI and the outcomes. **Results**: This study included 6207 individuals for CVD and 6386 individuals for all-cause mortality in CKM syndrome stages 0–3. In the fully adjusted models, TyGFI was significantly associated with increased risks of CVD (HR = 1.25, 95% CI: 1.18–1.33) and all-cause mortality (HR = 1.39, 95% CI: 1.18–1.63). Participants in the highest TyGFI quartile faced more risk of CVD incidence (HR = 2.02, 95% CI: 1.69–2.42) and death (HR = 2.67, 95% CI: 1.33–5.38) compared to those in the lowest quartile. RCS analysis revealed a significant non-linear association between TyGFI and CVD and a linear association with all-cause death. ROC analysis indicated that TyGFI had the stronger predictive ability for CVD. Mediation analysis showed that age mediated the effect of TyGFI on both CVD occurrence (mediation proportion = 9.81%) and mortality (mediation proportion = 32.45%). Subgroup and sensitivity analyses confirmed the robustness of the findings. **Conclusions**: The findings suggest that TyGFI is a strong predictor of CVD risk and mortality in individuals with CKM syndrome stages 0–3, and age may serve as a mediating factor. These findings hold important clinical significance for enhancing the early identification and prevention of cardiovascular and fatal events in middle-aged and elderly populations.

## 1. Introduction

Cardiovascular disease (CVD) remains a leading cause of death and disability worldwide. The number of prevalent CVD cases reached 626 million in 2023, twice the number reported in 1990 [1]. By 2050, cardiovascular deaths are expected to reach 35.6 million, which is a 73.4% increase in the crude mortality rate compared to 2025 [2]. Beyond demographic shifts, the sustained rise in CVD burden over decades is increasingly linked to metabolic risks, such as high body mass index (BMI) and elevated fasting blood glucose [3].

The American Heart Association (AHA) has introduced the concept of a cardiovascular-kidney-metabolic (CKM) syndrome, defining it as a progressive health disorder resulting from pathological interactions among CVD, chronic kidney disease (CKD), and metabolic disorders (including obesity, diabetes, etc.). The AHA has further classified CKM syndrome into five stages [4]. This health disorder, due to the inherent connection of multiple diseases, can significantly increase the risks of CVD events and mortality [5]. Data from the US Renal Data System 2020 show that the CVD burden is markedly greater in CKD patients, with prevalence increasing with CKD severity [6]. The molecular mechanisms underlying CKM syndrome may include hyperglycemia, insulin resistance (IR), oxidative stress, lipotoxicity, mitochondrial dysfunction, and chronic inflammation [7]. Recognizing the mechanisms underlying the interplay of CKM-related diseases facilitates a more comprehensive approach to the development of prevention and treatment strategies, rather than considering each condition in isolation. Notably, progression through CKM stages can be bidirectional, highlighting the potential for disease remission through targeted preventive measures [8].

IR, defined as decreased sensitivity to insulin leading to impaired glucose utilization, is closely linked to CVD. It contributes to atherosclerosis—a primary CVD risk factor—through mechanisms like dyslipidemia, inflammation, and endothelial dysfunction [9]. IR also has a bidirectional relationship with CKD. CKD can promote IR via chronic inflammation and oxidative stress, while IR can exacerbate renal impairment through mechanisms including sympathetic activation and sodium retention [10]. The triglyceride-glucose (TyG) index, derived from fasting triglyceride and glucose levels, is a robust surrogate marker for IR [11]. Studies show that a high TyG index is linked to a greater risk of CVD and death [12,13]. Beyond the TyG index, frailty is also closely associated with CVD and mortality [14]. Frailty is a state of increased vulnerability resulting from the progressive decline of physiological systems, typically characterized by reduced muscle mass and strength, limited physical activity, decreased endurance, and progressive multisystem functional decline [15]. It is a multidimensional concept encompassing physical, cognitive, social, psychological, and nutritional domains. Frailty is typically assessed using the frailty index (FI), which is calculated by accumulating multiple age-related health deficits, including comorbidities, physical and cognitive function, and mental health status [16]. Over a five-year follow-up period, frailty was strongly correlated with an elevated incidence of cardiovascular events such as mortality, myocardial infarction, stroke, and vascular disease [17]. The biological mechanisms linking frailty to CVD may involve shared physiological pathways, such as inflammation, metabolic dysfunction, and IR [18]. Fortunately, frailty is not an irreversible state. It is a dynamic process, and although findings suggest the existence of a point of irreversibility, early intervention for frailty syndrome may still yield benefits [19]. Studies have highlighted the importance of early identification of frailty (enhanced education), personalized interventions (such as dietary adjustments, exercise programs, and cognitive training), real-time monitoring and adjustment, and the establishment of multidisciplinary teams to address frailty in cardiovascular practice [20,21]. In fact, the TyG index is closely associated with frailty. A cohort study showed that IR is related to an increased risk of frailty, indicating that preventing or controlling IR can delay the occurrence of frailty [22]. The relationship between the TyG index and frailty may involve underlying mechanisms such as inflammation, oxidative stress, impaired protein synthesis, and dysregulation of metabolic pathways [23,24]. Therefore, we constructed the composite index TyG-frailty index (TyGFI) by multiplying TyG and FI (TyG × FI), based on the rationale that IR and frailty share common pathways, and this model construction may better reflect their combined effect.

Although there is an established association between TyG and frailty with cardiovascular risk and mortality, they are rarely evaluated together, particularly in patients with CKM syndrome stages 0–3. Recent biological studies have indicated that obesity promotes biological aging through chronic inflammation and oxidative stress, impairs endothelial function, and accelerates vascular aging, thereby increasing the risk of CVD [25]. A multi-cohort study also suggested that biological age acceleration partially mediates the association between obesity and cardiovascular events [26]. Meanwhile, frailty itself is a complicated age-related medical condition, and age-related reduction in muscle mass contributes to physical problems and metabolism regulation problems, which are also impairing insulin signals and thereby promoting IR [27]. Therefore, this study utilized data from the China Health and Retirement Longitudinal Study (CHARLS) to evaluate the associations of the TyGFI with CVD and mortality, separately, in the context of CKM syndrome stages 0–3 and further explored the role of age as a mediator in these associations. The findings of this study may contribute to a more comprehensive assessment of CVD risk and mortality, particularly in middle-aged and older adults with concurrent metabolic and renal disease burdens.

## 2. Materials and Methods

### 2.1. Study Design and Population

Data were obtained from the CHARLS, a nationally representative longitudinal survey that collects social, economic, and health information from Chinese adults aged 45 years and older through standardized questionnaires and clinical assessments [28]. The baseline survey was conducted between June 2011 and March 2012, involving 17,708 respondents, with follow-ups every two to three years (2013, 2015, 2018, and 2020). The study protocol adhered to the Declaration of Helsinki and was approved by the Peking University Institutional Review Board (IRB00001052-11015). Informed written consent was obtained from all participants before the commencement of data collection. More information is available on the CHARLS official website (http://charls.pku.edu.cn/en accessed on 30 July 2025).

**Figure 1:**
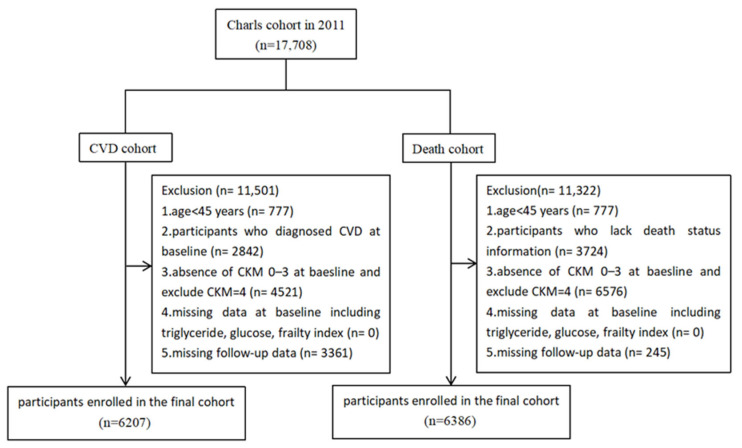
Flowchart of study population selection. CVD cohort: participants who were followed for incident CVD as the outcome. Death cohort: participants who were followed for all-cause mortality as the outcome.

The flowchart (Figure 1) illustrates the inclusion and exclusion criteria. From the baseline cohort of 17,708 individuals, we applied the following exclusion criteria: (1) age < 45 years at baseline; (2) missing data for CKM stages 0–3 or classification as CKM stage 4; (3) missing key variables for calculating TyGFI (triglycerides, fasting glucose, and frailty index); (4) lack of follow-up data; (5) for the CVD cohort: a baseline diagnosis of CVD (heart disease or stroke); (6) for the mortality cohort: missing information on vital status. Finally, 6207 and 6386 participants were included in the CVD and mortality cohorts, respectively, for subsequent analyses assessing the association of TyGFI with CVD incidence and all-cause mortality. The distribution of variables with missing data is shown in Table S1. Missing data were handled using multiple imputation to ensure the robustness of the results.

### 2.2. Data Collection

The variables gathered in this study included: Sociodemographic information, such as age, gender, marriage status, place of residence, smoking habits, drinking behavior, and sleeping time, were acquired through standard interviewer-administered questionnaires. Anthropometric and clinical measurements such as waist circumference (WC), height, weight, systolic blood pressure (SBP), diastolic blood pressure (DBP), and BMI were recorded by trained health care professionals following standardized procedures. Laboratory biomarkers, such as fasting plasma glucose, glycated hemoglobin (HbA1c), triglycerides (TG), total cholesterol (TC), high-density lipoprotein cholesterol (HDL-C), low-density lipoprotein cholesterol (LDL-C), C-reactive protein (CRP), serum creatinine (Scr), blood urea nitrogen (BUN), and estimated glomerular filtration rate (eGFR), were analyzed from venous blood samples that had been collected from township hospitals or the Chinese Center for Disease Control and Prevention after at least 8 h of fasting. Information about the physician-diagnosed illnesses (hypertension, diabetes, dyslipidemia, CVD, cancer, lung diseases, liver problems) and the current medicines used for these illnesses was also gathered. Diagnostic criteria for these conditions are detailed in Table S2.

The TyG index was calculated as Ln[TG (mg/dL) × Fasting Glucose (mg/dL)/2], which is commonly applied in metabolic cardiovascular research [29,30]. In our study, we constructed the FI according to the method proposed by Searle et al. [16]. To prevent circularity and guarantee the validity of the association analyses, items directly related to CVD (heart problems, stroke) were excluded. Our final FI model comprised 30 items covering chronic diseases, sensory function, activities of daily living, depressive symptoms, and cognitive function (Table S3). FI was calculated as the number of health deficits present divided by the total number of deficits assessed, with higher values indicating greater frailty [31]. The composite index TyGFI was constructed using a multiplicative model: TyGFI = TyG × FI. As employed in prior epidemiological analyses, this modeling approach can capture the potential interactive effects between metabolic dysregulation and physical-functional decline [32,33].

### 2.3. Definition of CKM Syndrome Stages 0–3

In accordance with the AHA scientific statement, CKM syndrome is categorized into the following stages: stage 0 comprises individuals without any CKM-related risk factors; stage 1 is defined by early metabolic disturbances such as overweight and/or dysfunctional obesity and/or impaired glucose tolerance; stage 2 includes established metabolic conditions, including individuals with diabetes, hypertension or hypertriglyceridemia, and CKD; and stage 3 indicates the presence of subclinical cardiovascular abnormalities, including a predicted high 10-year CVD risk or high-risk CKD [4,34]. Details can be found in Table S4.

### 2.4. Outcome Ascertainment

The primary outcomes were CVD incidence and all-cause mortality in individuals with CKM stages 0–3. Incident CVD cases (including CVD and stroke) were identified based on participants’ self-reported physician diagnoses during follow-up waves [35]. All-cause mortality data were obtained from the CHARLS exit interviews, verified through death certificates, medical records, or interviews with family members [28]. The CHARLS team employed rigorous standards for data recording and validation to ensure reliability [36].

### 2.5. Statistical Analysis

The Kolmogorov–Smirnov test is used for normality testing. Quantitative variables following a normal distribution are presented as mean ± standard deviation (SD), and group differences are compared using one-way analysis of variance (ANOVA). For quantitative variables not following a normal distribution, median and interquartile range are reported, and group differences are assessed using the Kruskal–Wallis test. Categorical variables are presented as frequencies and percentages, and comparisons are made using the chi-square test. To enhance the robustness of the analysis, TyGFI was examined both as a continuous and a categorical variable. Accordingly, study participants were also stratified into four groups based on TyGFI quartiles. Multivariable Cox proportional hazards models were constructed to assess the associations of TyGFI with incident CVD and all-cause mortality. The proportional hazards assumption was tested using Schoenfeld residuals. For covariates that violated the proportional hazards assumption, a log transformation was applied. A global Schoenfeld test *p*-value > 0.05 was considered indicative of no significant violation of the proportional hazards assumption. The results are presented as hazard ratios (HRs) with corresponding 95% confidence intervals (CIs). For the CVD cohort, three models were specified: The crude model was not adjusted, Model 1 was adjusted for age, gender, smoking status, alcohol consumption, sleep duration, WC, BMI, and weight, and Model 2 was further adjusted for LDL-C, HbA1c, total cholesterol, HDL-C, eGFR, hypertension, diabetes, and dyslipidemia on top of all the Model 1 covariates. Mortality analysis: Model 1 was adjusted for age, gender, marital status, smoking status, alcohol consumption, sleep duration, height, and weight. Model 2 also added CRP, total cholesterol, eGFR, cancer, hypertension, and diabetes. In order to reduce the possible multicollinearity among covariates, the VIF was calculated for each variable. Only variables that had a VIF of less than 5 were kept in the final multivariable models [37].

To explore potential non-linear associations between TyGFI and CVD occurrence as well as all-cause mortality, a restricted cubic spline (RCS) model was constructed with four knots placed at the 5th, 35th, 65th, and 95th percentiles of TyGFI. When a non-linear relationship was suggested, a two-piece Cox regression model was fitted and compared against a linear Cox model using the likelihood ratio test. Kaplan–Meier curves were plotted to examine the predictive value of TyGFI for CVD risk and mortality. Furthermore, to compare the predictive performance of different metabolic indicators for CVD and all-cause mortality, receiver operating characteristic (ROC) curve analysis was performed, and the area under the curve (AUC) was calculated. The DeLong test was used to assess the statistical significance of differences between AUCs derived from the same participants, with TyG-FI prespecified as the reference for all comparisons. The net reclassification improvement (NRI) and integrated discrimination improvement (IDI) were further used to compare the predictive value for CVD between TyG-FI and TyG. An NRI of 0 indicates no improvement in the new model, whereas an NRI > 0 indicates that the new model is better than the old model. For IDI, a value greater than 0 indicates better predictive performance of the new model, with larger values reflecting greater improvement. To further investigate potential causal relationships, subgroup analyses were performed based on the following variables: sex, age (45–60, ≥60 years), drinking status, smoking status, BMI (<24, 24–28, ≥28 kg/m^2^), hypertension, diabetes, dyslipidemia, and CKM stage 0–3. Interaction terms between TyGFI and each subgroup variable were incorporated into Cox regression models, which were adjusted for covariates included in Model 2. To avoid overadjustment, the respective subgroup variable itself was excluded from the covariate set. The results were visualized using forest plots. The CMAverse package in R was employed to perform mediation analysis, estimating the total effects, direct effects, indirect effects, the proportion mediated, and corresponding *p*-values to examine whether age mediates the associations between TyGFI and CVD risk and mortality [38]. This analysis was done through 1000 posterior simulations to find out the distribution of the mediation effect.

Sensitivity analyses were conducted to evaluate the robustness of the findings. First, missing data in this study were handled using multiple imputation for analysis. In addition, we also performed a complete-case analysis (excluding observations with missing data) as a validation analysis. Second, logistic regression models were employed as an alternative analytical approach to verify the consistency of the results. Third, a post hoc power analysis was performed using G*Power 3.1 to assess whether the study sample size was sufficient to detect the observed effects, with a power of ≥80% considered indicative of reliable conclusions [39]. Fourth, we constructed an additive model (TyG + FI) and compared the results with those from the original model. Fifth, to assess the potential impact of unmeasured confounding, we calculated the E-value for the HRs obtained from the Cox regression models. Finally, to account for the competing risk of death and the potential impact of follow-up duration on our findings, we constructed a Fine-Gray competing-risk model and re-analyzed the data using 2015 as the outcome assessment point. Due to the exploratory nature of this study, which was designed to generate rather than test hypotheses, no adjustment for multiple comparisons was applied [40]. All analyses were performed using R version 4.2.3, and a *p*-value < 0.05 was considered statistically significant.

## 3. Results

### 3.1. Baseline Characteristics

Finally, the CVD group consisted of 6207 people (2819 men and 3388 women; average age: 57.51 ± 8.41 years), and the mortality group comprised 6386 individuals (2916 men and 3470 women; average age: 57.76 ± 8.57 years). From Table 1, we can see that in the CVD group, people who developed new CVD have higher average numbers for age, WC, BMI, weight, LDL-C, CRP, HbA1c, fasting glucose, TC, TG, SBP, DBP, TyGFI, TyG, and FI. They also had more chances of having high blood pressure, diabetes, and dyslipidemia (*p* < 0.05). On the other hand, those who got CVD had lower eGFR, HDL-C, and sleep time (*p* < 0.05). Table S5 indicates that in the mortality group, people who passed away had greater average ages, CRP, Scr, fasting blood glucose, SBP, DBP, TyGFI, TyG, and FI than those who survived, and they were more likely to have hypertension, diabetes, and cancer (*p* < 0.05). On the contrary, deceased persons had smaller numbers for height, weight, LDL-C, TC, TG, eGFR, and sleep duration (*p* < 0.05). Baseline characteristics based on TyGFI quartiles are given in Tables S6 and S7.

### 3.2. Association of TyGFI with CVD and Mortality in CKM Syndrome Patients

As shown in Table 2, multivariate Cox regression analysis revealed that among individuals at CKM stages 0–3, TyGFI was significantly and positively associated with the incidence of CVD (Model 2: HR 1.25, 95% CI 1.18–1.33, *p* < 0.0001). Compared to the lowest quartile (Q1) of TyGFI, CVD incidence was significantly higher in Q2, Q3, and Q4. In model 2, the risk of CVD was increased by 23% in Q2 (HR 1.23, 95% CI 1.02–1.48, *p* = 0.028), by 54% in Q3 (HR 1.54, 95% CI 1.29–1.85, *p* < 0.0001), and by 102% in Q4 (HR 2.02, 95% CI 1.69–2.42, *p* < 0.0001) relative to Q1 (*p* for trend < 0.0001). After adjusting for FI covariates, the risk increase in Q4 compared to Q1 was 87% (HR 1.87, 95% CI 1.40–2.49, *p* < 0.0001), which was lower than the risk seen with TyGFI alone (Table S8). In contrast, after covariate adjustment, TyG did not show any significant difference between Q4 and Q1 (Table S9). For all-cause mortality, every one-unit rise in continuous TyGFI was connected to a 39% greater danger (Model 2: HR 1.39, 95% CI 1.18–1.63, *p* < 0.0001). Stratified analysis found that the mortality was 167% higher in Q4 compared to Q1 (Model 2: HR 2.67, 95% CI 1.33–5.38, *p* = 0.006), but there was no significant relationship for Q2 and Q3 (*p* > 0.05), indicating a threshold effect of TyGFI (Table S10). The proportional hazards assumption was satisfied (global P_CVD = 0.62, P_mortality = 0.13) (Tables S11 and S12). All VIFs were below the conventional threshold of 5 (Tables S13 and S14).

### 3.3. RCS and Threshold Effect Analysis

**Figure 2:**
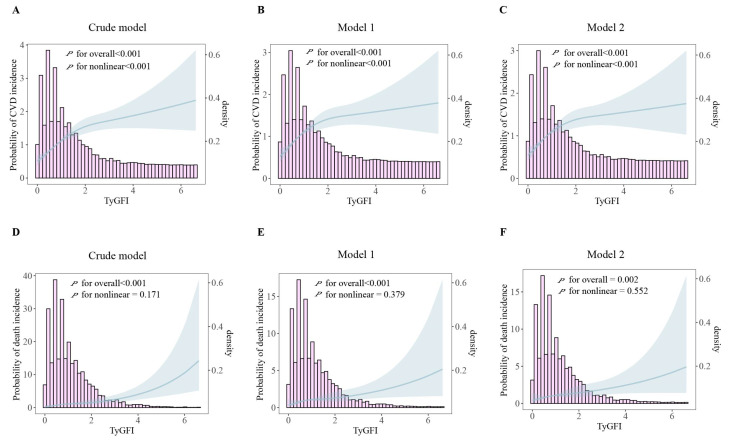
RSC showing the connection between TyGFI, CVD incidence and all-cause death events. (**A**–**C**) CVD. Crude model: unadjusted for covariates; Model 1: Adjust for: age, gender, smoking status, alcohol consumption, sleep duration, WC, BMI, and weight; Model 2: Adjusted for: LDL-C, HbA1c, TC, HDL-C, eGFR, hypertension, diabetes, and dyslipidemia in addition to all Model 1 covariates. (**D**–**F**) All-cause mortality. Crude model: unadjusted for covariates; Model 1: Adjust for: age, gender, marital status, smoking status, alcohol consumption, sleep duration, height, and weight; Model 2: Adjusted for: CRP, TC, eGFR, cancer, hypertension, and diabetes in addition to all Model 1 covariates.

We performed RCS analysis to evaluate potential nonlinear relationships between TyGFI and the risk of incident CVD as well as all-cause mortality. As illustrated in Figure 2, a nonlinear association was observed between TyGFI and CVD incidence (HR 1.60, 95% CI 1.40–1.83, *p* < 0.0001). Given this nonlinearity, we conducted a threshold effect analysis using a two-piecewise Cox regression model, which identified a significant inflection point at TyGFI = 1.006. Below this threshold, each unit increase in TyGFI was significantly associated with a higher risk of CVD (Model 2: HR 2.389, 95% CI 1.738–3.283, *p* < 0.0001). Above this level, TyGFI still had a significant association with increased CVD risk, but with a lower effect size (Model 2: HR 1.163, 95% CI 1.060–1.275, *p* = 0.001). The log-likelihood ratio test showed that the two-piecewise model fit better than the linear model, indicating a non-linear dose–response relationship between TyGFI and CVD risk (*p* < 0.0001) (Table S15). On the other hand, there was no significant nonlinear relationship found between TyGFI and all-cause mortality (*p* = 0.552).

### 3.4. Kaplan–Meier Survival Curves

**Figure 3:**
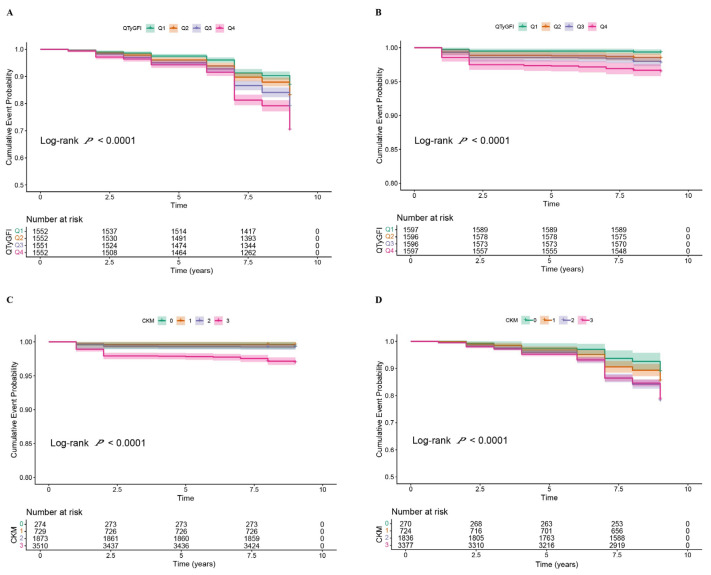
Kaplan–Meier survival curves. (**A**) CVD-free survival rates for the different TyGFI classification groups (Q1, Q2, Q3 and Q4). (**B**) All-cause mortality for the different TyGFI classification groups (Q1, Q2, Q3 and Q4). (**C**) CVD-free survival rates for the different CKM staging groups (0–3). (**D**) All-cause mortality for the different CKM staging groups (0–3).

Kaplan–Meier survival analysis was performed based on quartiles, which showed that participants in the highest TyGFI quartile (Q4) were at the highest risk of CVD occurrence and mortality over time, while those in the lowest quartile (Q1) had the lowest risk. Log-rank test indicated a significant difference among quartiles (*p* < 0.001) (Figure 3). Moreover, Kaplan–Meier curves stratified according to CKM stages 0–3 showed an increase in cumulative CVD incidence and mortality with rising CKM stage (*p* < 0.001).

### 3.5. Comparative Predictive Performance of TyG, FI, and TyGFI

In terms of predicting CVD risk, TyGFI showed the best predictive power (AUC = 0.61, 95% CI: 0.59–0.63), which was slightly better than FI (AUC = 0.60, 95% CI: 0.59–0.62); meanwhile, TyG had the worst predictive ability (AUC = 0.56, 95% CI: 0.54–0.57). Likewise, with respect to all-cause mortality, TyGFI (AUC = 0.66, 95% CI: 0.62–0.71) and FI (AUC = 0.67, 95% CI: 0.62–0.72) showed similar predictive capabilities, whereas TyG once more revealed inferior predictive capability (AUC = 0.54, 95% CI: 0.49–0.59) (Figure S1). DeLong’s test suggested that TyGFI had a much better ability to predict CVD compared to both FI and TyG (*p* < 0.001). We compared the baseline model plus TyGFI with the same baseline model plus TyG using NRI and IDI. Based on the NRI and IDI estimates, the composite index TyGFI demonstrated better predictive ability (NRI%: 20.44, 95% CI 14.29–26.60, *p* < 0.001; IDI%: 0.93, 95% CI 0.62–1.23, *p* < 0.001).

### 3.6. Subgroup Analyses

**Figure 4:**
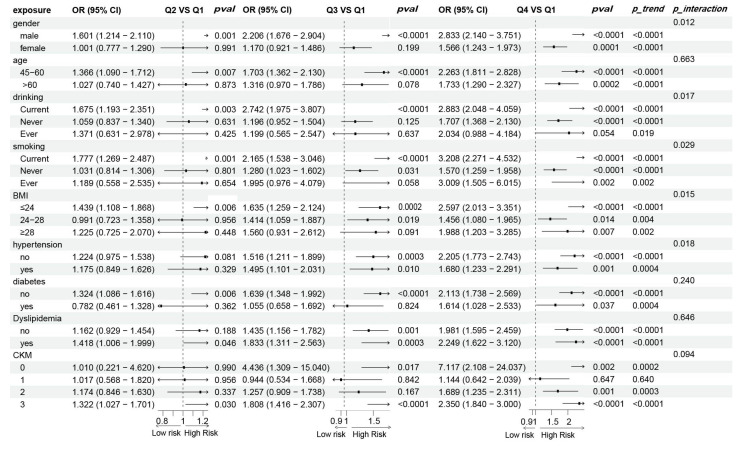
Subgroup analyses of the association between TyGFI and CVD incidence.

To further investigate the associations between TyGFI and CVD or all-cause mortality, subgroup and interaction analyses were conducted using covariates including age, sex, smoking, alcohol consumption, BMI, diabetes, hypertension, dyslipidemia, and CKM syndrome stages 0–3. In the CVD cohort, sex-stratified analysis revealed that in males, higher TyGFI quartiles were consistently associated with increased CVD risk (*p* < 0.0001), with a clear dose–response relationship: Q2 vs. Q1 HR 1.601, Q3 vs. Q1 HR 2.206, Q4 vs. Q1 HR 2.833 (*p* < 0.0001). In females, only Q4 was significantly associated with increased CVD risk (HR 1.566, 95% CI 1.243–1.973, *p* = 0.001). Additionally, men had a much greater CVD risk than women, and there was a significant interaction between sex (*p* = 0.012). Smoking status (*p* = 0.029), alcohol consumption (*p* = 0.017), hypertension (*p* = 0.036), and BMI (*p* = 0.015) also showed significant interaction effects. Current smokers (Q4 vs. Q1 HR 3.208), current drinkers (Q4 vs. Q1 HR 2.883), non-hypertensive individuals (Q4 vs. Q1 HR 2.205), and those with BMI < 24 (Q4 vs. Q1 HR 2.597) had a higher risk of CVD. On the other hand, there were no significant interactions for age, diabetes, dyslipidemia, or CKM stage (Figure 4, Table S16). In the mortality group, only the participants who did not have dyslipidemia showed a significant positive relationship with all-cause mortality (Q4 vs. Q1 HR 4.524, 95% CI 1.750–11.695, *p* = 0.002), and there was an interaction effect for dyslipidemia (*p* = 0.048) (Figure S2, Table S17).

### 3.7. Mediation of Age in the Relationship Between TyGFI and CVD or Death

**Figure 5:**
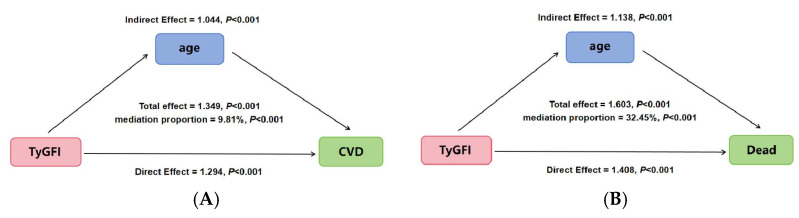
Mediation analysis of age in the association between TyGFI and outcomes. (**A**) Mediation analysis with CVD as the outcome. Adjust for: age, gender, smoking status, alcohol consumption, sleep duration, WC, BMI, weigh, LDL-C, HbA1c, TC, HDL-C, eGFR, hypertension, diabetes, and dyslipidemia. (**B**) Mediation analysis with all-cause mortality as the outcome. Adjust for: age, gender, marital status, smoking status, alcohol consumption, sleep duration, height, weight, CRP, TC, eGFR, cancer, hypertension, and diabetes.

Mediation analysis showed that age had a significant mediating effect on the relationship between TyGFI and CVD (estimate = 1.349, 95% CI 1.273–1.427, *p* < 0.001). Direct effect was 1.293 (*p* < 0.001), indirect effect was 1.044 (*p* < 0.001), and proportion mediated was 9.81% (*p* < 0.001). Similarly, in the mortality cohort, TyGFI was also found to have an indirect impact on all-cause mortality via age (estimate = 1.603, 95% CI 1.317–1.855, *p* < 0.001), with a direct effect of 1.408 (*p* < 0.001), an indirect effect of 1.138 (*p* < 0.001), and a mediation proportion of 32.45% (*p* < 0.001) (Figure 5).

### 3.8. Sensitivity Analyses

To assess the stability of the findings, several sensitivity analyses were carried out. First, we conducted a complete case analysis as a validation analysis, and the results were consistent with those of the primary analysis (Tables S18 and S19). Second, logistic regression analyses were carried out instead of Cox proportional hazards models, and the results were still consistent with the main analysis (Tables S20 and S21). Third, piecewise Cox regression models and subgroup analyses were used, both showing a consistent relationship between TyGFI and the outcomes of interest (Tables S16 and S17). Fourth, the post hoc power analysis showed that both the CVD cohort (n = 6207, HR = 2.02) and the mortality cohort (n = 6386, HR = 2.67) had statistical power over 99% (Table S22). Fifth, an additive model was constructed for regression analysis. The results showed no significant association between the additive model and CVD or all-cause mortality (Tables S23 and S24). Sixth, we calculated the E-values for the HRs. The E-value for the association between TyGFI and CVD was 1.815, and that for mortality was 2.119. These results indicate that unmeasured confounding is unlikely to substantially change the findings. In addition, we constructed a Fine-Gray competing-risk model to account for the competing risk of death. The results were consistent with our primary findings (SHR = 1.25, 95% CI: 1.19–1.33, *p* < 0.001). Finally, we re-analyzed the data using 2015 as the outcome assessment point (Tables S25 and S26), and the results from this 4-year follow-up analysis were consistent with those from the 9-year follow-up.

## 4. Discussion

This study utilized data from the nationally representative CHARLS cohort, including participants with CKM syndrome stages 0–3. Using Cox proportional hazards models, it was the first study to evaluate the association between the novel composite index TyGFI and the risks of incident CVD and all-cause mortality in individuals with CKM syndrome stages 0–3. The findings revealed that even after adjusting for sociodemographic characteristics, anthropometric measures, laboratory biomarkers, and other factors, higher levels of TyGFI remained significantly associated with increased risks of CVD and all-cause mortality. A threshold-driven nonlinear relationship was observed between TyGFI and CVD incidence: below the threshold of 1.006, the risk of CVD increased significantly; whereas above this threshold, the association attenuated. In contrast, TyGFI showed a continuous linear association with all-cause mortality. In the fully adjusted model, each unit increase in continuous TyGFI was associated with a 39% increase in mortality risk. Subgroup analysis revealed that the impact of TyGFI on CVD risk was more pronounced for males, drinkers, smokers, those without hypertension, and individuals with low BMI. Mediation analysis indicated that age mediated the effect of TyGFI on both CVD and mortality. Overall, these results support TyGFI as a reliable biomarker for predicting CVD and mortality risk in patients with CKM syndrome stages 0–3, and provide critical evidence for risk stratification in this population.

Extensive research has established that the TyG index as a reliable surrogate marker of IR, demonstrating significant positive associations with CVD risk and mortality. For instance, a cohort study reported that each unit increase in the highest quartile of the TyG index was associated with an 80% higher CVD risk and a 60% increase in all-cause mortality [41,42]. Similarly, several prospective cohort studies have shown that the FI can predict the onset of CVD and death, with improved frailty status linked to a 38% reduction in CVD risk, while a 0.1 increase in FI corresponded to a 68% higher all-cause mortality [43,44]. Both the TyG index, reflecting IR, and the FI, indicating multisystem physiological decline, are central factors in cardiometabolic vulnerability. The combined TyGFI index integrates these two dimensions, potentially better capturing their synergistic effects. Consistent with this, our study found that for predicting CVD risk, TyGFI showed the highest predictive utility. However, it is important to acknowledge that some studies have reported conflicting or nuanced results. For example, regarding the TyG index, the meta-analysis found no significant association between the TyG index and all-cause or cardiovascular mortality [45]. Regarding the frailty index component, a prospective study conducted in the Brazilian community-dwelling elderly found that the FI was not capable of predicting mortality in that population [46]. These discrepancies may be attributable to differences in study populations, follow-up durations, and adjustment for confounding variables, highlighting the potential complexity of the relationship when metabolic and frailty components are combined and warranting further investigation.

Although TyGFI has demonstrated predictive value for cardiovascular events, the specific mechanisms underlying its association with CVD and all-cause mortality in individuals with CKM syndrome stages 0–3 remain unclear [31]. Based on existing evidence, we propose that the following pathways may be involved. IR activates inflammatory signaling pathways such as Jun N-terminal kinase (JNK) and IKK/NF-κB, and promotes the production of pro-inflammatory cytokines such as IL-1β, IL-6, TNF-α, and MCP-1 [47,48]. It also leads to dyslipidemia characterized by elevated triglycerides, low HDL, and high VLDL, which may be related to altered expression of insulin receptor substrate-1 (IRS-1) and AKT serine/threonine kinase 2 [49,50]. Dyslipidemia is a well-known early event in the development of atherosclerotic CVD. And also, IR can speed up atherosclerosis via endothelial dysfunction, brought about by blocked nitric oxide creation, activation of the MAPK pathway, vasoconstriction, and hypertrophy of vascular smooth muscle cells [51,52]. At the same time, frailty promotes the release of inflammatory factors (such as IL-6, IL-8, CRP, and TNF-α) and oxidative stress [53]. This chronic inflammation and immune activation may further impair vascular endothelial function, thereby contributing to CVD progression. Although the molecular interaction between IR and frailty is not fully understood, studies suggest that IR may lead to frailty by impairing skeletal muscles glucose handling and reducing microvascular blood flow, resulting in loss of muscle mass and strength [54]. This process may also involve chronic inflammation and age-related hormone reductions such as insulin-like growth factor 1 (IGF-1) [55,56]. Supporting this, Tian et al., using public databases and Mendelian randomization analysis, found that the TyG index was independently associated with frailty progression and physical decline, with a causal link between higher TyG levels and increased frailty risk [57]. Beyond these established pathways, emerging evidence suggests that mitochondrial dysfunction may serve as a common mechanistic link between IR and frailty. IR is associated with impaired mitochondrial biogenesis and reduced oxidative capacity in skeletal muscle, leading to ectopic fat accumulation and further IR [58]. Meanwhile, frailty-related mitochondrial dysfunction contributes to reduced energy production, increased oxidative stress, and accelerated cellular senescence [59]. Furthermore, epigenetic modifications, including DNA methylation and histone modifications, may mediate the long-term effects of metabolic dysregulation on frailty and CVD outcomes [60,61]. Chronic hyperglycemia and inflammation can induce persistent epigenetic changes that alter gene expression profiles related to inflammation, metabolism, and aging [62]. While these mechanisms remain largely speculative, they represent important directions for future research. In summary, IR and frailty share overlapping mechanisms and exhibit mutually reinforcing synergistic effects, thereby amplifying the risk of CVD and mortality. This mechanistic convergence provides a strong rationale for the TyGFI composite index [13,43].

Through subgroup analysis, we observed that the impact of TyGFI on CVD risk was more pronounced in individuals without hypertension. This suggests that IR and frailty may constitute an independent pathway leading to vascular damage and atherosclerosis, which operates independently of elevated blood pressure. In contrast, patients with pre-existing hypertension are more likely to have coexisting risk factors such as obesity, poor dietary habits, smoking, and alcohol consumption [63]. Under such conditions, the incremental prognostic value provided by the TyGFI index may be partially attenuated. Conversely, among normotensive individuals, the metabolic derangements and physiologic changes captured by TyGFI become key indicators for identifying those at risk for subclinical CVD. Therefore, assessment of TyGFI can facilitate earlier lifestyle interventions or therapeutic management for populations at high risk of CVD. Regarding sex differences, our analysis indicated that TyGFI had a stronger predictive value for CVD in male patients. This may be attributable to the higher prevalence of abdominal obesity and greater visceral adipose tissue in males, which is closely linked to IR [64]. Furthermore, estrogen is known to exert protective effects on insulin sensitivity and cardiovascular health in females, potentially mitigating some of the adverse effects associated with IR and thus attenuating the predictive power of TyGFI in women [65]. Consequently, TyGFI may reflect a more direct path to CVD in males. In the context of primary prevention of CVD, greater emphasis should be placed on screening and managing IR in male populations.

Our mediation analysis demonstrated that age mediated 9.81% and 32.45% of the associations between TyGFI and the risks of CVD and mortality, respectively, indicating that age serves as a key mediator linking metabolic dysregulation to cardiovascular pathology and death. Previous studies have shown that with advancing age, elevated levels of myostatin contribute to the decline in muscle mass and strength, leading to frailty [66]. Concurrently, aging itself is associated with increased IR. While the etiology of IR is multifactorial, population aging has been identified as a contributing factor, supported by epidemiological studies showing higher prevalence rates of IR and type 2 diabetes in older adults [67]. Although aging is irreversible, our findings underscore the importance of proactive health management in the elderly. Older individuals should be encouraged to adopt lifestyle modifications, including dietary adjustments, regular physical activity, and timely pharmacotherapy when indicated, to mitigate their risk of CVD and mortality.

To enhance the robustness and clinical significance of our findings, this study employed multiple methods, including complete-case analysis, validation via logistic regression, RCS modeling, Kaplan–Meier curves, and subgroup analyses. Our results revealed a non-linear relationship between TyGFI and CVD risk. Specifically, above the threshold of 1.006, the increase in CVD risk per unit increment in TyGFI was markedly attenuated. The precise mechanism underlying this nonlinear association remains unclear; however, we hypothesize that extreme frailty may lead to a cachectic state characterized by low body weight and malnutrition-related hypolipidemia, which could alter the dose–response relationship between the TyG index and vascular risk [68]. The observed nonlinear association suggests that early preventive interventions are particularly warranted for individuals whose TyGFI levels remain below the threshold, as focusing resources and health education on this group may yield greater benefits. This approach aligns well with the principles of primary prevention and early screening. The study population comprised individuals in CKM stages 0–3, representing the preclinical or early clinical phase of CVD progression, a stage where the identification of early predictive factors is crucial [5]. Our findings highlight the substantial potential of TyGFI in enabling risk-stratified preventive strategies for CVD.

### 4.1. Strengths of the Research

Our study has some strengths. Firstly, we used nationally representative cohort data of Chinese older adults, focusing on CKM stages 0–3 patients to enable timely interventions. Secondly, to the best of our knowledge, it is the first time that the TyGFI has been used to assess CVD incidence and all-cause mortality in this particular group of patients, which makes it innovative and clinically relevant. Thirdly, the study included 6207 and 6386 participants in the CVD and mortality cohorts, respectively, with statistical power greater than 99% for both outcomes. Fourthly, we used two different regression models, covariate adjustment, and subgroup analyses to make sure our results were robust. RCS analysis showed that there may be a nonlinear relationship and a threshold effect between TyGFI and CVD risk. Finally, mediation analysis showed that age was a mediator for the effect of TyGFI on both CVD and mortality, which improved the comprehensiveness of our conclusion and limitations.

### 4.2. Limitations and Future Perspectives

Despite the rigorous methodology and multiple validation approaches, this study has several limitations. First, CVD, mortality, and CKM progression are dynamic processes. But the data for TyGFI and other variables originated from baseline surveys and prescribed follow-up periods, so it is difficult to know how metabolic and bodily functions change over time, possibly missing some important details about how things moved around. Future studies will explore the impact of cumulative TyGFI exposure on CVD incidence and all-cause mortality. Second, since we relied on the CHARLS database, our sample consisted only of middle-aged and older Chinese adults, which could restrict the generalizability of our findings to other age strata and populations. Validation in different kinds of groups is needed if possible. Third, as regards CKM staging, due to data limitations, we did not use the most recent CKM-specific risk prediction models but rather the traditional Framingham 10-year CVD risk score. This may affect the accuracy of the staging. Fourth, outcomes were mostly based on self-reported diagnosis. Although the CHARLS database has demonstrated satisfactory accuracy, this approach may introduce recall or misclassification bias (e.g., inaccurate recall or undiagnosed asymptomatic cases), which could attenuate the true associations and lead to an underestimation of the effect sizes and predictive performance of our models. Future validation using objective clinical endpoints is therefore highly warranted. Fifth, even after adjusting for many covariates, we still cannot completely exclude residual confounding due to unmeasured factors. Future studies could improve causal inference through Mendelian randomization or use more cohorts and prediction algorithms. Sixth, although our mediation analysis identified age as a potential mediator to highlight the importance of early intervention, the interpretation of age as a mediator remains conceptually debatable because age is an irreversible variable. Future studies are needed to further clarify this conceptual issue. Lastly, the new predictor TyGFI utilized in this study is generated from a multiplication equation. Supported by epidemiological evidence and our own findings, this method is still experimental and needs more testing in real-world settings.

## 5. Conclusions

This study evaluated the association between an innovative predictor, TyGFI, and the risks of CVD and mortality in individuals at CKM syndrome stages 0–3, using data from the CHARLS database. The results showed that elevated TyGFI levels were significantly associated with an increased risk of CVD and all-cause mortality, and age may serve as a mediating factor. This strong association remained significant after adjusting for multiple confounding factors. Subgroup analysis and RCS analysis confirmed the robustness of these findings and suggested a potential non-linear relationship. These outcomes indicate that TyGFI may serve as a reliable biomarker with significant predictive value for CVD and mortality in middle-aged and elderly populations at CKM syndrome stages 0–3.

## Figures and Tables

**Table 1:** Baseline characteristics of the study individuals with and without CVD.

Variable	Overall (n = 6207)	Non-CVD (n = 4954)	CVD (n = 1253)	*p* Value
Age, year	57.00 (51.00, 63.00)	56.00 (50.00, 52.00)	58.00 (53.00, 64.00)	<0.001
Gender, n (%)				0.003
Female	3388 (54.58)	2657 (53.63)	522 (41.66)	
Male	2819 (45.42)	2297 (46.37)	1080 (86.19)	
Marital, n (%)				0.875
married	5339 (86.02)	4259 (85.97)	1080 (86.19)	
unmarried	868 (13.98)	695 (14.03)	173 (13.81)	
Residence, n (%)				0.668
rural	4200 (67.67)	3359 (67.80)	841 (67.12)	
urban	2007 (32.33)	1595 (32.20)	412 (32.88)	
Smoking, n (%)				0.013
Current	1893 (30.50)	1540 (31.09)	353 (28.17)	
Ever	444 (7.15)	334 (6.74)	110 (8.78)	
Never	3870 (62.35)	3080 (62.17)	393 (31.36)	
Drinking, n (%)				0.022
Current	2106 (33.93)	1713 (34.58)	393 (31.36)	
Ever	448 (7.22)	340 (6.86)	108 (8.62)	
Never	3653 (58.85)	2901 (58.56)	752 (60.02)	
Sleep, hour	7.00 (5.00, 8.00)	7.00 (5.00, 8.00)	6.00 (5.00, 8.00)	0.019
WC, cm	84.00 (74.40, 91.00)	83.40 (77.00, 90.20)	87.00 (79.80, 94.40)	<0.001
BMI, kg/m^2^	23.12 (20.91, 25.67)	22.96 (20.79, 25.35)	23.92 (21.55, 26.71)	<0.001
Height, m	1.58 (1.52, 1.64)	1.58 (1.52, 1.64)	1.57 (1.52, 1.64)	0.394
Weight, kg	57.70 (51.10, 65.30)	57.30 (50.80, 64.70)	59.60 (52.30, 68.00)	<0.001
LDL-c, mg/dL	114.43 (93.94, 137.63)	113.66 (93.17, 136.08)	117.91 (96.65, 141.50)	<0.001
TC, mg/dL	190.98 (167.78, 215.34)	189.82 (167.40, 214.56)	194.46 (170.88, 219.98)	<0.001
TG, mg/dL	103.54 (74.34, 152.22)	101.78 (72.57, 148.68)	111.51 (80.54, 161.07)	<0.001
HDL-c, mg/dL	49.87 (40.98, 60.31)	50.26 (40.98, 60.70)	48.71 (40.21, 58.38)	<0.001
CRP, mg/dL	0.95 (0.52, 1.97)	0.90 (0.50, 1.89)	1.17 (0.60, 2.28)	<0.001
Glucose, mg/dL	102.06 (94.14, 112.41)	101.70 (93.96, 111.60)	103.68 (95.40, 115.20)	<0.001
HBA1C, %	5.10 (4.90, 5.40)	5.10 (4.90, 5.40)	5.20 (4.90, 5.50)	<0.001
Scr, mg/dL	0.76 (0.64, 0.87)	0.76 (0.64, 0.87)	0.76 (0.66, 0.87)	0.787
UA, mg/dL	4.24 (3.55, 5.09)	4.25 (3.56, 5.08)	4.23 (3.53, 5.11)	0.759
BUN, mg/dL	15.13 (12.52, 18.12)	15.15 (12.52, 18.21)	15.10 (12.55, 17.87)	0.565
eGFR, mL/min/1.73 m^2^	95.96 (85.92, 103.07)	96.38 (86.22, 103.54)	94.46 (84.42, 100.95)	<0.001
SBP, mmHg	125.00 (112.50, 139.00)	123.50 (112.00, 137.50)	130.00 (117.00, 145.00)	<0.001
DBP, mmHg	74.00 (66.50, 82.50)	73.50 (66.00, 81.50)	76.50 (68.50, 85.00)	<0.001
TyG	8.58 (8.21, 9.01)	8.55 (8.19, 9.00)	8.68 (8.32, 9.11)	<0.001
FI	0.09 (0.05, 0.17)	0.09 (0.05, 0.15)	0.12 (0.08, 0.21)	<0.001
TyGFI	0.79 (0.42, 1.45)	0.75 (0.41, 1.36)	1.06 (0.63, 1.82)	<0.001
Hypertension, n (%)				<0.001
No	4399 (70.87)	3621 (73.09)	778 (62.09)	
Yes	1808 (29.13)	1333 (26.91)	475 (37.91)	
Diabetes, n (%)				<0.001
No	5263 (84.79)	4239 (85.57)	1024 (81.72)	
Yes	944 (15.21)	715 (14.43)	229 (18.28)	
Dyslipidemia, n (%)				<0.001
No	4385 (70.65)	3598 (72.63)	787 (62.81)	
Yes	1822 (29.35)	1356 (27.37)	466 (37.19)	
Cancer, n (%)				0.445
No	6165 (99.32)	4918 (99.27)	1247 (99.52)	
Yes	42 (0.68)	36 (0.73)	6 (0.48)	
Lung diseases, n (%)				<0.001
No	5746 (92.57)	4626 (93.38)	1120 (89.39)	
Yes	461 (7.43)	328 (6.62)	133 (10.61)	
Liver diseases, n (%)				0.003
No	6037 (97.26)	4834 (97.58)	1203 (96.01)	
Yes	170 (2.74)	120 (2.42)	50 (3.99)	
CKM, n (%)				<0.001
0	270 (4.35)	240 (4.84)	30 (2.39)	
1	724 (11.66)	619 (12.49)	105 (8.38)	
2	1836 (29.58)	1436 (28.99)	400 (31.92)	
3	3377 (54.41)	2659 (53.67)	718 (57.30)	

*TyGFI* triglyceride-glucose and frailty index, *TyG* triglyceride-glucose, *FI* frailty index, *CKM* cardiovascular–kidney–metabolic, *CVD* cardiovascular disease, *BMI* body mass index, *CRP* C-reactive protein, *WC* waist circumference, *HbA1c* hemoglobin A1c, *Scr* serum creatinine, *BUN* blood urea nitrogen, *eGFR* estimated glomerular filtration ratio, *TG* triglycerides, *TC* total cholesterol, *LDL-C* low-density lipoprotein cholesterol, *HDL-C* high-density lipoprotein cholesterol, *SBP* systolic blood pressure, *DBP* diastolic blood pressure.

**Table 2:** Multivariate Cox regression for the correlation between TyGFI and CVD risk.

TyGFI	Crude Model		Model 1		Model 2	
	HR (95% CI)	*p*	HR (95% CI)	*p*	HR (95% CI)	*p*
CVD incidence						
Continuous	1.35 (1.28, 1.42)	<0.0001	1.28 (1.21, 1.35)	<0.0001	1.25 (1.18, 1.33)	<0.0001
Categories						
Q1	Ref					
Q2	1.32 (1.10, 1.59)	0.003	1.28 (1.06, 1.54)	0.009	1.23 (1.02, 1.48)	0.028
Q3	1.67 (1.40, 1.99)	<0.0001	1.60 (1.34, 1.92)	<0.0001	1.54 (1.29, 1.85)	<0.0001
Q4	2.47 (2.10, 2.92)	<0.0001	2.16 (1.81, 2.57)	<0.0001	2.02 (1.69, 2.42)	<0.0001
*p* for trend		<0.0001		<0.0001		<0.0001

Crude model: unadjusted for covariates; Model 1: age, gender, smoke, drink, sleep, WC, BMI, weight; Model 2: age, gender, smoke, drink, sleep, WC, BMI, weight, LDL-c, HBA1C, TC, HDL-c, eGFR, hypertension, diabetes, dyslipidemia.

## Data Availability

The CHARLS dataset used in this study is publicly available from the official website (https://charls.pku.edu.cn) (accessed on 30 July 2025). Users can access and download the data according to the platform’s guidelines, following registration and approval.

## Supplementary Materials

The following supporting information can be downloaded at: https://www.mdpi.com/article/10.3390/jcm15114156/s1, Figure S1: Time-dependent ROC curves of the TyGFI, TyG indices and FI for predicting CVD onset and all-cause mortality onset. (A) CVD. (B) All-cause mortality. Figure S2: Subgroup analyses of the association between TyGFI and all-cause mortality. Table S1: Distribution of variables with missing data. Table S2: Specific definitions of various diseases. Table S3: Methods for constructing the frailty index in CHARLS. Table S4: Methods for evaluating CKM stages 0–3. Table S5: Baseline characteristics of the study individuals with and without all-cause death. Table S6: Baseline characteristics of the study individuals in CVD incidence. Table S7: Baseline characteristics of the study individuals in mortality. Table S8: Multivariate cox regression for the correlation between FI and CVD incidence. Table S9: Multivariate cox regression for the correlation between TyG and CVD incidence. Table S10: Multivariate cox regression for the correlation between TyGFI and all-cause mortality. Table S11: Cox models using the Schoenfeld residuals test in CVD cohort. Table S12: Cox models using the Schoenfeld residuals test in mortality cohort. Table S13: Multicollinearity evaluation of predictor variables through variance inflation factor (VIF) analysis in CVD cohort. Table S14: Multicollinearity evaluation of predictor variables through variance inflation factor (VIF) analysis in mortality cohort. Table S15: Threshold effect analysis of TyGFI on CVD incidence using a two-piecewise linear regression model. Table S16: Association between TyGFI and CVD stratified by sex, age, drink status, smoke status, BMI, hypertension, dyslipidemia, diabetes and CKM stages. Table S17: Association between TyGFI and all-cause mortality stratified by sex, age, drink status, smoke status, BMI, hypertension, dyslipidemia, diabetes and CKM stages. Table S18: Multivariate cox regression for the correlation between TyGFI and CVD risk: complete-case analysis. Table S19: Multivariate cox regression for the correlation between TyGFI and all-cause mortality: complete-case analysis. Table S20: Multivariate logistic regression for the correlation between TyGFI and CVD risk. Table S21: Multivariate logistic regression for the correlation between TyGFI and all-cause mortality. Table S22: Post-Hoc Power Calculations to assess the efficacy of observed associations. Table S23: Multivariate cox regression for the correlation between TyGFI (additive model) and CVD incidence. Table S24: Multivariate cox regression for the correlation between TyGFI (additive model) and all-cause mortality. Table S25: Multivariate cox regression for the correlation between TyGFI and CVD incidence during a four-year follow-up. Table S26: Multivariate cox regression for the correlation between TyGFI and all-cause mortality during a four-year follow-up.

## Abbreviations

The following abbreviations are used in this manuscript:

TyGFI	triglyceride-glucose and frailty index
FI	frailty index
CKM	cardiovascular–kidney–metabolic
CHARLS	China Health and Retirement Longitudinal Study
CVD	Cardiovascular disease
BMI	body mass index
CRP	C-reactive protein
WC	waist circumference
HbA1c	Hemoglobin A1c
Scr	serum creatinine
BUN	blood urea nitrogen
eGFR	estimated glomerular filtration ratio
TG	triglycerides
TC	total cholesterol
LDL-C	low-density lipoprotein cholesterol
HDL-C	high-density lipoprotein cholesterol
SBP	systolic blood pressure
DBP	diastolic blood pressure
HR	Hazard ratios
95% CI	95% confidence interval
AUC	Area under the curve
RCS	Restricted cubic spline
