# Supplementary material for: Association of Triglyceride-Glucose-Frailty Index with Cardiovascular Disease and All-Cause Mortality Incidence in Individuals with Cardiovascular-Kidney-Metabolic Syndrome Stages 0–3: A Nationwide Prospective Cohort Study
Source: J Clin Med. 2026 May 28;15(11):4156. doi: 10.3390/jcm15114156 (PMC13258764; doi:10.3390/jcm15114156)
Supplement: Supplementary file 1 [file jcm-15-04156-s001.zip › jcm-4252302-supplementary.pdf]

Abbreviations

TyGFI: triglyceride-glucose and frailty index
TyG: triglyceride-glucose
FI: frailty index
CKM: cardiovascular–kidney–metabolic
CHARLS: China Health and Retirement Longitudinal Study
CVD: Cardiovascular disease
BMI: body mass index
CRP: C-reactive protein
WC: waist circumference
HbA1c: Hemoglobin A1c
Scr: serum creatinine
BUN: blood urea nitrogen
eGFR: estimated glomerular filtration ratio
TG: triglycerides
TC: total cholesterol
LDL-C: low density lipoprotein cholesterol
HDL-C: high density lipoprotein cholesterol
SBP: systolic blood pressure
DBP: diastolic blood pressure
HR: Hazard ratios
95%*CI:* 95% confidence interval
AUC: Area under the curve
RCS: Restricted cubic spline

Fig S1. Time-dependent ROC curves of the TyGFI, TyG indices and FI for predicting CVD onset and all-cause mortality onset. (A) CVD. (B) All-cause mortality

Fig S2. Subgroup analyses of the association between TyGFI and all-cause mortality

Table S1. Distribution of variables with missing data

Table S2. Specific definitions of various diseases

Table S3. Methods for constructing the frailty index in CHARLS

Table S4. Methods for evaluating CKM stages 0-3

Table S5. Baseline characteristics of the study individuals with and without all-cause death

Table S6. Baseline characteristics of the study individuals in CVD incidence

Table S7. Baseline characteristics of the study individuals in mortality

Table S8. Multivariate cox regression for the correlation between FI and CVD incidence

Table S9. Multivariate cox regression for the correlation between TyG and CVD incidence

Table S10. Multivariate cox regression for the correlation between TyGFI and all-cause mortality

Table S11. Cox models using the Schoenfeld residuals test in CVD cohort.

Table S12. Cox models using the Schoenfeld residuals test in mortality cohort.

Table S13. Multicollinearity evaluation of predictor variables through variance inflation factor (VIF) analysis in CVD cohort.

Table S14. Multicollinearity evaluation of predictor variables through variance inflation factor (VIF) analysis in mortality cohort.

Table S15. Threshold effect analysis of TyGFI on CVD incidence using a two-piecewise linear regression model

Table S16. Association between TyGFI and CVD stratified by sex, age, drink status, smoke status, BMI, hypertension, dyslipidemia, diabetes and CKM stages

Table S17. Association between TyGFI and all-cause mortality stratified by sex, age, drink status, smoke status, BMI, hypertension, dyslipidemia, diabetes and CKM stages

Table S18. Multivariate cox regression for the correlation between TyGFI and CVD risk: complete-case analysis

Table S19. Multivariate cox regression for the correlation between TyGFI and all-cause mortality: complete-case analysis

Table S20. Multivariate logistic regression for the correlation between TyGFI and CVD risk

Table S21. Multivariate logistic regression for the correlation between TyGFI and all-cause mortality

Table S22. Post-Hoc Power Calculations to assess the efficacy of observed associations

Table S23. Multivariate cox regression for the correlation between TyGFI (additive model) and CVD incidence.

Table S24. Multivariate cox regression for the correlation between TyGFI (additive model) and all-cause mortality.

Table S25. Multivariate cox regression for the correlation between TyGFI and CVD incidence during a four-year follow-up

Table S26. Multivariate cox regression for the correlation between TyGFI and all-cause mortality during a four-year follow-up

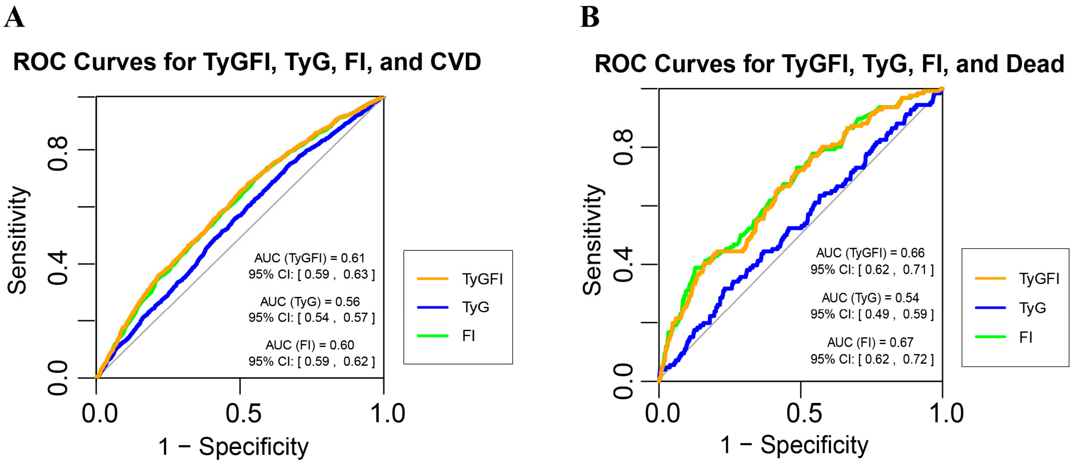

Fig. S1 Time-dependent ROC curves of the TyGFI, TyG indices and FI for predicting CVD onset and all-cause mortality onset. (A) CVD. (B) All-cause mortality.

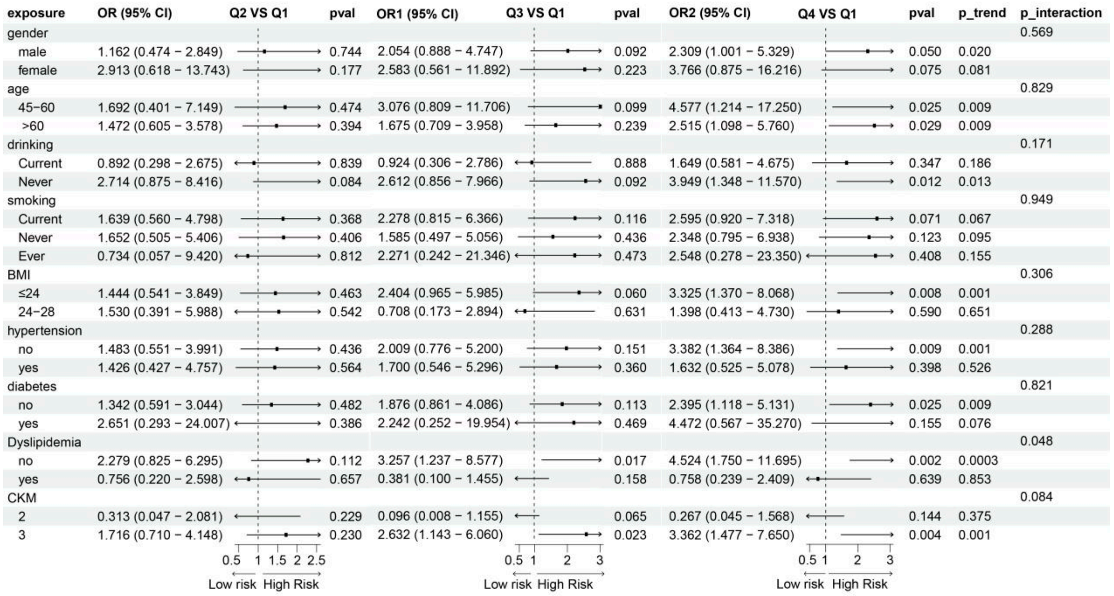

Fig. S2 Subgroup analyses of the association between TyGFI and all-cause mortality.

Table S1. Distribution of variables with missing data

Variables	CVD incidence		morality	
	Number of Missing	Proporation	Number of Missing	Proportion
Smoking	7	0.11%	8	0.13%
Drinking	3	0.05%	3	0.05%
Sleep	66	1.06%	73	1.14%
WC	99	1.59%	103	1.61%
LDL_c	10	0.16%	10	0.17%
BUN	1	0.02%	1	0.02%
SBP	15	0.24%	17	0.27%
DBP	14	0.23%	16	0.25%
Hypertension	10	0.16%	12	0.19%
Dyslipidemia	1	0.02%	1	0.02%
Cancer	9	0.14%	9	0.14%
Lung diseases	5	0.08%	5	0.08%
Liver disease	14	0.23%	15	0.23%

WC: Waist circumference; LDL-C: low density lipoprotein cholesterol; BUN: blood urea nitrogen; SBP: Systolic blood pressure; DBP: diastolic blood pressure

Table S2. Specific definitions of various diseases

CVD	Heart attack, coronary heart disease, angina, congestive heart failure, stroke or other heart problems
Diabetes	Fasting blood glucose ≥ 125 mg/dL or HbA1c ≥ 6.5% or self-reported diagnosis of diabetes, use of insulin, or oral hypoglycemic agents
Hypertension	SBP ≥130 mmHg or DBP ≥80 mmHg or self-reported diagnosis of hypertension or use of antihypertensive medications
Dyslipidemia	TG ≥ 200 mg/dL or TC ≥ 240 mg/dL or LDL-C ≥ 160 mg/dL or self-reported diagnosis of dyslipidemia or use of lipid-lowering medications
Cancer	Cancer or malignant tumor (excluding minor skin cancers)
Lung diseases	Chronic lung diseases, such as chronic bronchitis , emphysema (excluding tumors, or cancer)
Liver diseases	Liver diseases (except fatty liver, tumors, and cancer)

Table S3. Methods for constructing the frailty index in CHARLS

No	Description of the item	Cut-off value
1	Self-reported physician diagnosed hypertension	Yes = 1, No = 0
2	Self-reported physician diagnosed diabetes	Yes = 1, No = 0
3	Self-reported physician diagnosed asthma	Yes = 1, No = 0
4	Self-reported physician diagnosed cancer	Yes = 1, No = 0
5	Self-reported physician diagnosed arthritis	Yes = 1, No = 0
6	Self-reported physician diagnosed chronic lung disease	Yes = 1, No = 0
7	Self-reported physician diagnosed any emotional, nervous, or psychiatric problems	Yes = 1, No = 0
8	Self-reported physician diagnosed memory-related disease	Yes = 1, No = 0
9	Self-reported vision problems	Yes = 1, No = 0
10	Self-reported hearing problems	Yes = 1, No = 0
11	Self-reported general health status	Very poor or poor = 1, Very good, good, or fair = 0
12	Difficulty with dressing	Yes = 1, No = 0
13	Difficulty with bathing or showering	Yes = 1, No = 0
14	Difficulty with eating	Yes = 1, No = 0
15	Difficulty with getting in and out of bed	Yes = 1, No = 0
16	Difficulty with using the toilet	Yes = 1, No = 0
17	Difficulty with managing money	Yes = 1, No = 0
18	Difficulty with taking medication	Yes = 1, No = 0
19	Difficulty with shopping for groceries	Yes = 1, No = 0
20	Difficulty with preparing meals	Yes = 1, No = 0
21	Difficulty with doing housework	Yes = 1, No = 0
22	Mobility: difficulty with walking 100 yards or one block	Yes = 1, No = 0
23	Mobility: difficulty with getting up from a chair after sitting for long periods	Yes = 1, No = 0

24	Mobility: difficulty with climbing several flights of stairs without resting	Yes = 1, No = 0
25	Mobility: difficulty with lifting or carrying weights over 10 pounds/jins	Yes = 1, No = 0
26	Mobility: difficulty with picking up a coin from the table	Yes = 1, No = 0
27	Mobility: difficulty with stooping, kneeling, or crouching	Yes = 1, No = 0
28	Mobility: difficulty with reaching arms above shoulder level	Yes = 1, No = 0
29	Depression: CESD-10 questionnaire	CESD-10 >10 = 1, ≤10 = 0
30	Cognition: (memory test score + orientation test score) / 14	Continuous variable, ranging from 0 to 1

Depression was assessed by the Center for Epidemiologic Studies Depression Scale (CESD). In the CHARLS, CESD-10 was used, and the total score ranged from 0 to 30. A higher score indicated more severe depressive symptoms.

The memory score was the average of words which were not recalled in the immediate and delayed word recall tasks. The memory score ranged from 0 to 10. The orientation test comprised 4 questions about the day of the week, the month, the date of the month, and the year. One point was given for each wrong answer, and the range was from 0 to 4.

Table S4. Methods for evaluating CKM stages 0-3

CKM stages	Threshold for CKM conditions
Stages 0	All criteria are met：<br>① BMI< 23 kg/m2<br>② Waist circumference <80/90 cm in female/male<br>③ Fasting blood glucose < 100 mg/dL and HbA1c < 5.7% and without self-reported diagnosis of diabetes, use of insulin, or oral hypoglycemic agents.<br>④ SBP <130 mm Hg and DBP <80 mm Hg without self-reported diagnosis of hypertension or use of antihypertensive medications.<br>⑤ HDL-C <50/40 mg/dL in female/male<br>⑥ TG < 150 mg/dL<br>⑦ eGFR ≥ 60 ml/min/1.73m2 and without self-reported diagnosis of CKD<br>⑧ No Subclinical CVD and clinical CVD
Stages 1	Any of the three criteria is met:<br>① Overweight/obesity<br>② Abdominal obesity<br>③ Prediabetes<br>All criteria are met：<br>（1）SBP <130 mmHg and DBP <80 mmHg without self-reported diagnosis of hypertension or use of antihypertensive medications.<br>（2）HDL-C <50/40 mg/dL in female/male<br>（3）TG < 150 mg/dL<br>（4）eGFR ≥ 60 ml/min/1.73m2 and without self-reported diagnosis of CKD<br>（5）No Subclinical CVD and clinical CVD
Stages 2	Any of the five criteria is met:<br>① Hypertriglyceridemia<br>② Hypertension<br>③ Diabetes<br>④ metabolic syndrome<br>⑤ eGFR：30-60 ml/min/1.73m2 and/or with self-reported diagnosis of CKD<br>All criteria are met：<br>① No Subclinical CVD and clinical CVD
Stages 3	Any of the two criteria is met:<br>① eGFR<30 ml/min/1.73m2<br>② Subclinical CVD<br>Any of the eight criteria is met:<br>① Overweight/obesity<br>② Abdominal obesity<br>③ Prediabetes<br>④ Hypertriglyceridemia<br>⑤ Hypertension<br>⑥ diabetes

	⑦ metabolic syndrome<br>⑧ eGFR：30-60 ml/min/1.73m2 and/or with self-reported diagnosis of CKD<br>The criterion is met：<br>① No clinical CVD

Table S5. Baseline characteristics of the study individuals with and without all-cause death

Variable	Overall (n=6386)	Non-dead (n=6260)	Dead (n=126)	*P* value
Age, year	57.00 (51.00,63.00)	57.00 (51.00,63.00)	69.50 (60.25,75.00)	<0.001
Gender, n (%)				0.007
Female	3470 (54.34)	3417 (54.58)	53 (42.06)	
Male	2916 (45.66)	2843 (45.42)	73 (57.94)	
Marital, n (%)				<0.001
married	5464 (85.56)	5383 (85.99)	81 (64.29)	
unmarried	922 (14.44)	877 (14.01)	45 (35.71)	
Residence, n (%)				0.948
rural	4316 (67.59)	4230 (67.57)	86 (68.25)	
urban	2070 (32.41)	2030 (32.43)	40 (31.75)	
Smoking, n (%)				<0.001
Current	1975 (30.93)	1921 (30.69)	54 (42.86)	
Ever	462 (7.23)	444 (7.09)	18 (14.29)	
Never	3949 (61.84)	3895 (62.22)	54 (42.86)	
Drinking, n (%)				0.004
Current	2161 (33.84)	2120 (33.87)	41 (32.54)	
Ever	472 (7.39)	453 (7.24)	19 (15.08)	
Never	3753 (58.77)	3687 (58.90)	66 (52.38)	
Sleep, hour	6.50 (5.00,8.00)	7.00 (5.00,8.00)	6.00 (4.63,8.00)	0.027
WC, cm	84.00 (77.30,91.10)	84.00 (77.40,91.10)	83.45 (75.00,91.43)	0.141
BMI, kg/m2	23.11 (20.88,25.66)	23.13 (20.92,25.67)	21.04 (18.79,24.71)	<0.001
Height, m	1.58 (1.52,1.64)	1.58 (1.52,1.64)	1.56 (1.49,1.64)	0.048
Weight, kg	57.60 (51.00,65.20)	57.70 (51.10,65.30)	51.95 (43.95,63.30)	<0.001
LDL-c, mg/dl	114.43 (93.94,137.24)	114.63 (93.94,137.63)	104.77 (85.54,131.44)	0.015
TC, mg/dl	190.98 (167.78,215.34)	190.98 (167.78,215.34)	182.09 (158.60,212.05)	0.019
TG, mg/dl	103.54 (74.34,151.34)	103.54 (74.34,152.22)	92.93 (72.57,130.09)	0.017
HDL-c, mg/dl	49.87 (40.98,60.31)	49.87 (40.98,60.31)	48.90 (41.75,59.83)	0.982
CRP, mg/dl	0.97 (0.52,2.02)	0.96 (0.52,1.97)	2.16 (0.82,7.24)	<0.001
Glucose, mg/dl	102.06 (94.14,112.50)	102.06 (94.14,112.50)	103.95 (91.89,117.86)	0.335
HBA1C, %	5.10 (4.90,5.40)	5.10 (4.90,5.40)	5.20 (4.83,5.50)	0.253
Scr, mg/dl	0.76 (0.64,0.87)	0.76 (0.64,0.87)	0.80 (0.70,0.94)	0.001
UA, mg/dl	4.25 (3.55,5.10)	4.24 (3.55,5.09)	4.45 (3.56,5.45)	0.150
BUN, mg/dl	15.13 (12.55,18.12)	15.13 (12.52,18.12)	15.36 (12.82,18.35)	0.595
eGFR, mL/min/1.73m^2	95.69 (85.57,102.97)	95.86 (85.79,103.07)	87.04 (68.89,95.73)	<0.001
SBP, mmHg	125.00 (113.00,139.50)	125.00 (112.50,139.50)	134.50 (118.00,148.25)	<0.001
DBP, mmHg	74.00 (66.50,82.50)	74.00 (66.50,82.50)	74.50 (64.25,83.00)	0.487
TyG	8.57 (8.21,9.01)	8.58 (8.21,9.02)	8.50 (8.13,8.97)	0.144
FI	0.09 (0.05,0.17)	0.09 (0.05,0.17)	0.15 (0.09,0.27)	<0.001

**Variable**	**Overall (n=6386)**	**Non-dead (n=6260)**	**Dead (n=126)**	***P* value**
TyGFI	0.81 (0.43,1.47)	0.80 (0.42,1.46)	1.23 (0.75,2.28)	<0.001
Hypertension, n (%)				0.001
No	4499 (70.45)	4427 (70.72)	72 (57.14)	
Yes	1887 (29.55)	1833 (29.28)	54 (42.86)	
Diabetes, n (%)				0.005
No	5406 (84.65)	5311 (84.84)	95 (75.40)	
Yes	980 (15.35)	949 (15.16)	31 (24.60)	
Dyslipidemia, n (%)				0.144
No	4516 (70.72)	4419 (70.59)	97 (76.98)	
Yes	1870 (29.28)	1841 (29.41)	29 (23.02)	
Cancer, n (%)				<0.001
No	6339 (99.26)	6218 (99.33)	121 (96.03)	
Yes	47 (0.74)	42 (0.67)	5 (3.97)	
Lung diseases, n (%)				<0.001
No	5890 (92.23)	5794 (92.56)	96 (76.19)	
Yes	496 (7.77)	466 (7.44)	30 (23.81)	
Liver diseases, n (%)				0.265
No	6210 (97.24)	6090 (97.28)	120 (95.24)	
Yes	176 (2.76)	170 (2.72)	6 (4.76)	
CKM, n (%)				<0.001
0	274 (4.29)	272 (4.35)	2 (1.59)	
1	729 (11.42)	726 (11.60)	3 (2.38)	
2	1873 (29.33)	1859 (29.70)	14 (11.11)	
3	3510 (54.96)	3403 (54.36)	107 (84.92)	

Table S6. Baseline characteristics of the study individuals in CVD incidence

Variable	Overall (n=6207)	Q1 (n=1552)	Q2 (n=1552)	Q3 (n=1551)	Q4 (n=1552)	*P* value
Age, year	57.00 (51.00, 63.00)	54.00 (48.00,60.00)	56.00 (50.00,62.00)	57.00 (51.00,63.00)	59.00 (54.00,66.00)	<0.001
Gender, n (%)						<0.001
Female	3388 (54.58)	718 (46.26)	763 (49.16)	897 (57.83)	1010 (65.08)	
Male	2819 (45.42)	834 (53.74)	789 (50.84)	654 (42.17)	542 (34.92)	
Marital, n (%)						<0.001
married	5339 (86.02)	1397 (90.01)	1355 (87.31)	1312 (84.59)	1275 (82.15)	
unmarried	868 (13.98)	155 (9.99)	197 (12.69)	239 (15.41)	277 (17.85)	
Residence, n (%)						<0.001
rural	4200 (67.67)	938 (60.44)	1006 (64.82)	1066 (68.73)	1190 (76.68)	
urban	2007 (32.33)	614 (39.56)	546 (35.18)	485 (31.27)	362 (23.32)	
Smoking, n (%)						<0.001
Current	1893 (30.50)	545 (35.12)	532 (34.28)	442 (28.50)	374 (24.10)	
Ever	444 (7.15)	88 (5.67)	122 (7.86)	116 (7.48)	118 (7.60)	
Never	3870 (62.35)	919 (59.21)	898 (57.86)	993 (64.02)	1060 (68.30)	
Drinking, n (%)						<0.001
Current	2106 (33.93)	576 (37.11)	581 (37.43)	507 (32.69)	442 (28.50)	
Ever	448 (7.22)	62 (4.00)	90 (5.80)	122 (7.86)	174 (11.22)	
Never	3653 (58.85)	914 (58.89)	881 (56.77)	922 (59.45)	936 (60.28)	
Sleep, hour	7.00 (5.00, 8.00)	7.00 (6.00,8.00)	7.00 (6.00,8.00)	6.00 (5.00,8.00)	6.00 (4.00,7.00)	<0.001
WC, cm	84.00 (74.40, 91.00)	83.20 (77.18,90.00)	83.00 (77.20,90.60)	84.80 (77.40,91.40)	85.10 (78.00,93.00)	<0.001
BMI, kg/m2	23.12 (20.91,25.67)	23.09 (21.09,25.14)	22.89 (20.89,25.51)	23.23 (20.90,25.84)	23.31 (20.76,26.34)	0.050
Height, m	1.58 (1.52,1.64)	1.60 (1.54,1.66)	1.59 (1.52,1.65)	1.57 (1.51,1.63)	1.55 (1.50,1.61)	<0.001
Weight, kg	57.70 (51.10,65.30)	58.70 (52.77,65.83)	57.95 (51.30,65.12)	57.30 (50.70,65.00)	56.60 (49.50,65.10)	<0.001
LDL-C, mg/dl	114.43 (93.94,137.63)	113.66 (93.94,134.15)	115.21 (93.94,139.18)	114.82 (92.78,136.86)	115.01 (94.72,139.56)	0.122
TC, mg/dl	190.98 (167.78,215.34)	187.50 (165.08,210.70)	191.37 (167.78,216.88)	190.59 (167.78,215.34)	194.07 (170.49,219.59)	<0.001
TG, mg/dl	103.54 (74.34,152.22)	92.93 (67.26,138.06)	104.43 (76.11,153.10)	106.20 (75.22,152.22)	111.95 (80.54,165.72)	<0.001
HDL-C, mg/dl	49.87 (40.98,60.31)	50.06 (40.98,60.70)	49.48 (40.59,59.92)	49.48 (40.98,60.12)	49.87 (40.98,59.92)	0.572
CRP, mg/dl	0.95 (0.52,1.97)	0.82 (0.47,1.73)	0.96 (0.53,1.95)	0.96 (0.53,2.05)	1.06 (0.56,2.23)	<0.001
Glucose, mg/dl	102.06 (94.14,112.41)	100.62 (93.24,109.26)	102.24 (94.32,112.32)	102.42 (93.87,113.49)	103.41 (95.22,116.10)	<0.001
HBA1C, %	5.10 (4.90,5.40)	5.10 (4.80,5.40)	5.10 (4.90,5.40)	5.10 (4.90,5.40)	5.20 (4.90,5.50)	<0.001
Scr, mg/dl	0.76 (0.64,0.87)	0.76 (0.66,0.88)	0.77 (0.66,0.89)	0.75 (0.64,0.86)	0.72 (0.63,0.85)	<0.001
UA, mg/dl	4.24 (3.55,5.09)	4.29 (3.59,5.19)	4.30 (3.62,5.10)	4.25 (3.55,5.08)	4.13 (3.45,4.97)	<0.001
BUN, mg/dl	15.13 (12.52,18.12)	14.96 (12.38,17.81)	15.10 (12.58,18.07)	15.10 (12.55,18.23)	15.29 (12.60,18.37)	0.104
eGFR, mL/min/1.73m^2	95.96 (85.92,103.07)	97.25 (87.96,104.77)	96.31 (85.67,102.97)	95.81 (85.56,102.99)	94.24 (83.83,101.10)	<0.001
SBP, mmHg	125.00 (112.50,139.00)	122.00 (112.00,134.50)	124.00 (112.50,138.00)	126.00 (112.50,142.00)	127.50 (114.00,143.50)	<0.001
DBP, mmHg	74.00 (66.50,82.50)	73.50 (66.00,81.00)	74.00 (66.50,82.00)	74.50 (66.50,83.00)	74.00 (67.00,83.00)	0.140
TyG	8.58 (8.21,9.01)	8.45 (8.09,8.87)	8.59 (8.24,9.03)	8.61 (8.23,9.02)	8.67 (8.32,9.13)	<0.001
FI	0.09 (0.05,0.17)	0.02 (0.01,0.04)	0.08 (0.05,0.08)	0.12 (0.11,0.15)	0.23 (0.19,0.30)	<0.001
CVD, n (%)						<0.001
No	4954 (79.81)	1348 (86.86)	1288 (82.99)	1226 (79.05)	1092 (70.36)	
Yes	1253 (20.19)	204 (13.14)	264 (17.01)	325 (20.95)	460 (29.64)	
Hypertension, n						<0.001

**Variable**	**Overall (n=6207)**	**Q1 (n=1552)**	**Q2 (n=1552)**	**Q3 (n=1551)**	**Q4 (n=1552)**	***P* value**
(%)						
No	4399 (70.87)	1239 (79.83)	1130 (72.81)	1040 (67.05)	990 (63.79)	
Yes	1808 (29.13)	313 (20.17)	422 (27.19)	511 (32.95)	562 (36.21)	
Diabetes, n (%)						<0.001
No	5263 (84.79)	1409 (90.79)	1349 (86.92)	1290 (83.17)	1215 (78.29)	
Yes	944 (15.21)	143 (9.21)	203 (13.08)	261 (16.83)	337 (21.71)	
Dyslipidemia, n (%)						<0.001
No	4385 (70.65)	1197 (77.13)	1099 (70.81)	1104 (71.18)	985 (63.47)	
Yes	1822 (29.35)	355 (22.87)	453 (29.19)	447 (28.82)	567 (36.53)	
Cancer, n (%)						0.002
No	6165 (99.32)	1549 (99.81)	1544 (99.48)	1540 (99.29)	1532 (98.71)	
Yes	42 (0.68)	3 (0.19)	8 (0.52)	11 (0.71)	20 (1.29)	
Lung_diseases, n (%)						<0.001
No	5746 (92.57)	1538 (99.10)	1475 (95.04)	1409 (90.84)	1324 (85.31)	
Yes	461 (7.43)	14 (0.90)	77 (4.96)	142 (9.16)	228 (14.69)	
Liver_diseases, n (%)						<0.001
No	6037 (97.26)	1528 (98.45)	1521 (98.00)	1502 (96.84)	1485 (95.75)	
Yes	170 (2.74)	24 (1.55)	31 (2.00)	49 (3.16)	66 (4.25)	
CKM, n (%)						<0.001
0	270 (4.35)	95 (6.12)	73 (4.70)	55 (3.55)	47 (3.03)	
1	724 (11.66)	224 (14.43)	157 (10.12)	187 (12.06)	156 (10.05)	
2	1836 (29.58)	404 (26.03)	456 (29.38)	476 (30.69)	500 (32.22)	
3	3377 (54.41)	829 (53.41)	866 (55.80)	833 (53.71)	849 (55.70)	

Table S7. Baseline characteristics of the study individuals in mortality

Variable	Overall (n=6386)	Q1 (n=1597)	Q2 (n=1596)	Q3 (n=1596)	Q4 (n=1597)	*P* value
Age, year	57.00 (51.00,63.00)	54.00 (48.00,60.00)	56.00 (51.00,62.00)	57.00 (51.00,63.25)	60.00 (54.00,67.00)	<0.001
Gender, n (%)						<0.001
Female	3470 (54.34)	735 (46.02)	789 (49.44)	907 (56.83)	1039 (65.06)	
Male	2916 (45.66)	862 (53.98)	807 (50.56)	689 (43.17)	558 (34.94)	
Marital, n (%)						<0.001
married	5464 (85.56)	1436 (89.92)	1396 (87.47)	1337 (83.77)	1295 (81.09)	
unmarried	922 (14.44)	161 (10.08)	200 (12.53)	259 (16.23)	302 (18.91)	
Residence, n (%)						<0.001
rural	4316 (67.59)	965 (60.43)	1037 (64.97)	1090 (68.30)	1224 (76.64)	
urban	2070 (32.41)	632 (39.57)	559 (35.03)	506 (31.70)	373 (23.36)	
Smoking, n (%)						<0.001
Current	1975 (30.93)	566 (35.44)	546 (34.21)	472 (29.57)	391 (24.48)	
Ever	462 (7.23)	91 (5.70)	127 (7.96)	121 (7.58)	123 (7.70)	
Never	3949 (61.84)	940 (58.86)	923 (57.83)	1003 (62.84)	1083 (67.81)	
Drinking, n (%)						<0.001
Current	2161 (33.84)	596 (37.32)	594 (37.22)	520 (32.58)	451 (28.24)	
Ever	472 (7.39)	63 (3.94)	95 (5.95)	130 (8.15)	184 (11.52)	
Never	3753 (58.77)	938 (58.74)	907 (56.83)	946 (59.27)	962 (60.24)	
Sleep, hour	6.50 (5.00,8.00)	7.00 (6.00,8.00)	7.00 (6.00,8.00)	6.00 (5.00,8.00)	6.00 (4.00,7.00)	<0.001
WC, cm	84.00 (77.30,91.10)	83.20 (77.00,90.00)	83.40 (77.20,91.00)	84.80 (77.28,91.50)	85.20 (77.90,93.00)	<0.001
BMI, kg/m2	23.11 (20.88,25.66)	23.07 (21.07,25.16)	22.93 (20.90,25.53)	23.22 (20.84,25.83)	23.27 (20.70,26.27)	0.157
Height, m	1.58 (1.52,1.64)	1.60 (1.54,1.66)	1.58 (1.52,1.65)	1.57 (1.51,1.63)	1.55 (1.50,1.61)	<0.001
Weight, kg	57.60 (51.00,65.20)	58.70 (52.70,65.80)	58.00 (51.10,65.30)	57.30 (50.57,65.00)	56.20 (49.10,65.00)	<0.001
LDL-C, mg/dl	114.43 (93.94,137.24)	113.66 (93.56,134.15)	115.59 (94.72,139.56)	114.43 (92.40,136.47)	114.43 (94.72,139.18)	0.075
TC, mg/dl	190.98 (167.78,215.34)	187.50 (165.08,211.08)	191.75 (168.17,217.27)	189.82 (167.01,214.66)	193.69 (170.10,219.59)	<0.001
TG, mg/dl	103.54 (74.34,151.34)	92.9 3(67.26,138.06)	106.20 (76.11,155.76)	104.43 (74.34,149.57)	110.63 (80.54,163.73)	<0.001
HDL-C, mg/dl	49.87 (40.98,60.31)	49.87 (40.98,60.70)	49.10 (40.59,59.92)	49.87 (41.27,60.31)	49.87 (40.98,59.92)	0.458
CRP, mg/dl	0.97 (0.52,2.02)	0.83 (0.47,1.73)	0.98 (0.53,1.96)	0.98 (0.53,2.16)	1.07 (0.56,2.28)	<0.001
Glucose, mg/dl	102.06 (94.14,112.50)	100.62 (93.24,109.26)	102.24 (94.50,112.68)	102.42 (93.96,112.86)	103.50 (95.22,116.64)	<0.001
HBA1C, %	5.10 (4.90,5.40)	5.10 (4.80,5.40)	5.10 (4.90,5.40)	5.10 (4.90,5.40)	5.20 (4.90,5.50)	<0.001
Scr, mg/dl	0.76 (0.64,0.87)	0.77 (0.66,0.89)	0.77 (0.66,0.89)	0.75 (0.64,0.87)	0.72 (0.63,0.85)	<0.001
UA, mg/dl	4.25 (3.55,5.10)	4.30 (3.60,5.20)	4.30 (3.61,5.11)	4.25 (3.57,5.10)	4.13 (3.43,4.97)	<0.001
BUN, mg/dl	15.13 (12.55,18.12)	15.01 (12.41,17.81)	15.04 (12.58,18.02)	15.10 (12.55,18.26)	15.29 (12.60,18.37)	0.114
eGFR, mL/min/1.73m^2	95.69 (85.57,102.97)	97.25 (87.94,104.68)	96.11 (85.64,102.86)	95.70 (85.05,102.97)	93.89 (83.31,100.98)	<0.001
SBP, mmHg	125.00 (113.00,139.50)	122.00 (112.00,134.50)	1124.50 (113.00,138.50)	126.00 (112.50,142.00)	128.00 (114.00,144.00)	<0.001
DBP, mmHg	74.00 (66.50,82.50)	73.50 (66.00,81.50)	74.00 (66.50,82.50)	74.50 (66.50,83.00)	74.00 (66.50,83.00)	0.163
TyG	8.57 (8.21,9.01)	8.45 (8.10,8.87)	8.60 (8.24,9.05)	8.59 (8.22,9.00)	8.67 (8.33,9.13)	<0.001
FI	0.09 (0.05,0.17)	0.02 (0.01,0.04)	0.08 (0.05,0.08)	0.12 (0.11,0.15)	0.23 (0.19,0.30)	<0.001
Dead, n (%)						<0.001
No	6260 (98.03)	1586 (99.31)	1572 (98.50)	1561 (97.81)	1541 (96.49)	
Yes	126 (1.97)	11 (0.69)	24 (1.50)	35 (2.19)	56 (3.51)	
Hypertension, n (%)						<0.001

**Variable**	**Overall (n=6386)**	**Q1 (n=1597)**	**Q2 (n=1596)**	**Q3 (n=1596)**	**Q4 (n=1597)**	***P* value**
No	4495 (70.52)	1273 (79.76)	1148 (72.11)	1067 (66.98)	1007 (63.21)	
Yes	1879 (29.48)	323 (20.24)	444 (27.89)	526 (33.02)	586 (36.79)	
Diabetes, n (%)						<0.001
No	5406 (84.65)	1451 (90.86)	1378 (86.34)	1335 (83.65)	1242 (77.77)	
Yes	980 (15.35)	146 (9.14)	218 (13.66)	261 (16.35)	355 (22.23)	
Dyslipidemia, n (%)						<0.001
No	4516 (70.72)	1230 (77.02)	1118 (70.05)	1150 (72.06)	1018 (63.74)	
Yes	1870 (29.28)	367 (22.98)	478 (29.95)	446 (27.94)	579 (36.26)	
Cancer, n (%)						0.001
No	6339 (99.26)	1593 (99.75)	1588 (99.50)	1584 (99.25)	1574 (98.56)	
Yes	47 (0.74)	4 (0.25)	8 (0.50)	12 (0.75)	23 (1.44)	
Lung diseases, n (%)						<0.001
No	5890 (92.23)	1582 (99.06)	1515 (94.92)	1438 (90.10)	1355 (84.85)	
Yes	496 (7.77)	15 (0.94)	81 (5.08)	158 (9.90)	242 (15.15)	
Liver diseases, n (%)						<0.001
No	6210 (97.24)	1572 (98.43)	1560 (97.74)	1548 (96.99)	1530 (95.80)	
Yes	176 (2.76)	25 (1.57)	36 (2.26)	48 (3.01)	67 (4.20)	
CKM, n (%)						<0.001
0	274 (4.29)	98 (6.14)	73 (4.57)	53 (3.32)	50 (3.13)	
1	729 (11.42)	227 (14.21)	159 (9.96)	190 (11.90)	153 (9.58)	
2	1873 (29.33)	419 (26.24)	471 (29.51)	478 (29.95)	505 (31.62)	
3	3510 (54.96)	853 (53.41)	893 (55.95)	875 (54.82)	889 (55.67)	

Table S8. Multivariate cox regression for the correlation between FI and CVD incidence

FI	Crude model		Model 1		Model 2	
	**95% CI**	***P***	**95% CI**	***P***	**95% CI**	***P***
CVD						
Continuous	12.60 (8.02, 19.80)	< 0.0001	8.17 (4.93, 13.53)	< 0.0001	7.50 (4.48, 12.54)	< 0.0001
Categories						
Q1	Ref					
Q2	1.38 (1.16, 1.65)	0.0003	1.35 (1.13, 1.62)	0.001	1.30 (1.08, 1.57)	0.006
Q3	1,63 (1.37, 1.94)	< 0.0001	1.59 (1.33, 1.89)	0.027	1.48 (1.21, 1.81)	0.0002
Q4	2.44 (2.07, 2.87)	< 0.0001	2.15 (1.81, 2.56)	< 0.0001	1.87 (1.40, 2.49)	< 0.0001
P for trend		< 0.0001		< 0.0001		< 0.0001

Crude model: unadjusted for covariates; Model 1: age, gender, smoke, drink, sleep, WC, BMI, weight; Model 2: age, gender, smoke, drink, sleep, WC, BMI, weight, LDL-C, HBA1C, TC, HDL-C, eGFR, hypertension, diabetes, dyslipidemia.

Table S9. Multivariate cox regression for the correlation between TyG and CVD incidence

**TyG**	**Crude model**		**Model 1**		**Model 2**	
	**95% CI**	***P***	**95% CI**	***P***	**95% CI**	***P***
CVD						
Continuous	1.26 (1.17, 1.37)	< 0.0001	1.12 (1.03, 1.22)	0.007	0.95 (0.83, 1.08)	0.435
Categories						
Q1	Ref					
Q2	1.31 (1.10, 1.55)	0.002	1.23 (1.04, 1.46)	0.020	1.19 (1.00, 1.42)	0.047
Q3	1.55 (1.31, 1.82)	< 0.0001	1.32 (1.11, 1.56)	0.001	1.21 (1.01, 1,45)	0.034
Q4	1.59 (1.35, 1.87)	< 0.0001	1.27 (1.07, 1.50)	0.007	0.95 (0.76, 1.18)	0.640
P for trend		< 0.0001		0.017		0.562

Crude model: unadjusted for covariates; Model 1: age, gender, smoke, drink, sleep, WC, BMI, weight; Model 2: age, gender, smoke, drink, sleep, WC, BMI, weight, LDL-C, HBA1C, TC, HDL-C, eGFR, hypertension, diabetes, dyslipidemia.

Table S10. Multivariate cox regression for the correlation between TyGFI and all-cause mortality

TyGFI	Crude model		Model 1		Model 2	
	**HR (95% CI)**	***P***	**HR (95% CI)**	***P***	**HR (95% CI)**	***P***
All-cause mortality						
Continuous	1.67 (1.46, 1.91)	< 0.0001	1.38 (1.18, 1.62)	< 0.0001	1.39 (1.18, 1.63)	< 0.0001
Categories						
Q1	Ref					
Q2	2.19 (1.10, 4,48)	0.031	1.74 (0.83, 3.67)	0.145	1.5 (0.71, 3.20)	0.291
Q3	3.21 (1.63, 6.31)	< 0.001	2.24 (1.09, 4.58)	0.027	1.88 (0.91, 3.88)	0.087
Q4	5.17 (2.71, 9.86)	< 0.0001	2.93 (1.46, 5.87)	0.003	2.67 (1.33, 5.38)	0.006
P for trend		< 0.0001		< 0.0001		< 0.0001

Crude model: unadjusted for covariates; Model 1: age, gender, marital, smoke, drink, sleep, height, weight; Model 2: age, gender, marital, smoke, drink, sleep, height, weight, CRP, TC, eGFR, cancer, hypertension, diabetes.

Table S11. Cox models using the Schoenfeld residuals test in CVD cohort

Variable	chisq	df	P value
TyGFI	3.02e-01	1	0.58
age	2.04e+00	1	0.15
gender	1.76e-01	1	0.68
smoking	3.16e-01	2	0.85
drinking	1.98e-01	2	0.91
sleep	3.44e-01	1	0.56
Waist_circumference	9.91e-01	1	0.32
BMI	2.53e+00	1	0.11
weight	1.08e+00	1	0.30
LDL_c	7.16e+00	1	0.01
HBA1C	1.97e-05	1	0.99
TC	5.56e+00	1	0.02
HDL_c	2.84e+00	1	0.09
eGFR	1.48e+00	1	0.22
hypertension	1.33e-01	1	0.72
diabetes	1.73e+00	1	0.19
Dyslipidemia	2.10e-03	1	0.96
GLOBAL	1.66e+01	19	0.62

Table S12. Cox models using the Schoenfeld residuals test in mortality cohort

Variable	chisq	df	P value
TyGFI	2.62	1	0.43
age	0.71	1	0.40
gender	0.03	1	0.87
marital	0.72	1	0.40
smoking	0.50	2	0.78
drinking	0.42	2	0.81
sleep	0.63	1	0.43
height	4.33	1	0.04
weight	0.01	1	0.96
CRP	2.56	1	0.11
TC	0.88	1	0.35
eGFR	0.28	1	0.60
Cancer	1.38	1	0.24
hypertension	0.01	1	0.94
diabetes	1.32	1	0.25
GLOBAL	23.66	17	0.13

Table S13. Multicollinearity evaluation of predictor variables through variance inflation factor (VIF) analysis in CVD cohort.

Feature	VIF_TyGFI	VIF_TyG	VIF_FI
age	1.264	1.243	1.265
gender	1.613	1.604	1.612
smoking	1.210	1.210	1.210
drinking	1.111	1.108	1.111
sleep	1.031	1.011	1.031
Waist_circumference	1.272	1.272	1.272
BMI	1.051	1.050	1.051
weight	1.435	1.438	1.435
LDL_c	1.844	2.435	1.842
HBA1C	1.330	1.329	1.330
TC	2.105	1.705	2.105
HDL_c	1.180	2.859	1.180
eGFR	1.170	1.629	1.170
hypertension	1.046	1.170	1.046
diabetes	1.314	1.043	1.314
Dyslipidemia	1.280	1.313	1.280
Predictor variables	1.087	1.299	1.081

Table S14. Multicollinearity evaluation of predictor variables through variance inflation factor (VIF) analysis in mortality cohort.

Feature	VIF_TyGFI	VIF_TyG	VIF_FI
age	1.274	1.253	1.275
gender	1.756	1.749	1.755
marital	1.024	1.024	1.024
smoking	1.210	1.209	1.210
drinking	1.104	1.101	1.104
sleep	1.030	1.011	1.031
height	1.493	1.493	1.493
weight	1.242	1.271	1.242
CRP	1.006	1.006	1.006
TC	1.026	1.069	1.026
eGFR	1.172	1.172	1.172
Cancer	1.004	1.003	1.004
hypertension	1.045	1.044	1.045
diabetes	1.023	1.110	1.019
Predictor variables	1.089	1.197	1.086

Table S15. Threshold effect analysis of TyGFI on CVD incidence using a two-piecewise linear regression model

variable	Crude Model		Model 1		Model 2	
standard cox regression	1.78 (1.57, 2.02)	<0.0001	1.65 (1.45, 1.89)	<0.0001	1.60 (1.40, 1.83)	<0.0001
Log-likelihood value	-10807.14		-10579.52		-10541.29	
two-piecewise linear regression	threshold = 1.006		threshold = 1.006		threshold = 1.006	
Log-likelihood value	-10749.78		-10525.90		-10487.19	
TyGFI < threshold	2.63 (1.75, 3,97)	<0.0001	2,12 (1.19, 3.78)	0.0104	2.39 (1.74, 3.28)	<0.0001
TyGFI ⩾ threshold	1.21 (1.12, 1.31)	<0.0001	1.17 (1.08, 1.26)	<0.0001	1.16 (1.06, 1.27)	0.0014
p for Log-likelihood ratio	<0.0001		<0.0001		<0.0001	

Crude model: unadjusted for covariates; Model 1: age, gender, smoke, drink, sleep, WC, BMI, weight; Model 2: age, gender, smoke, drink, sleep, WC, BMI, weight, LDL-C, HBA1C, TC, HDL-C, eGFR, hypertension, diabetes, dyslipidemia.

Table S16. Association between TyGFI and CVD stratified by sex, age, drink status, smoke status, BMI, hypertension, dyslipidemia, diabetes and CKM stages

character	Q1	Q2	p	Q3	p	Q4	p	p for trend	p for interaction
Sex									
male	ref	1.601 (1.214, 2.110)	<0.0001	2.206 (1.676, 2.904)	<0.0001	2.833 (2.140, 3.751)	<0.0001	<0.0001	0.012
female	ref	1.001 (0.777, 1.290)	0.991	1.170 (0.921, 1.486)	0.199	1.566 (1.243, 1.973)	0.0001	<0.0001	
Age									
45-60	ref	1.366 (1.090, 1.712)	0.007	1.703 (1.362, 2.130)	<0.0001	2.263 (1.811, 2.828)	<0.0001	<0.0001	0.663
>60	ref	1.027 (0.740, 1.427)	0.873	1.316 (0.970, 1.786)	0.078	1.733 (1.290, 2.327)	0.0002	<0.0001	
Drink status									
current	ref	1.675 (1.193, 2.351)	0.003	2.742 (1.975, 3.807)	<0.0001	2.883 (2.048, 4.059)	<0.0001	<0.0001	0.017
never	ref	1.059 (0.837, 1.340)	0.631	1.196 (0.952, 1.504)	0.125	1.707 (1.368, 2.130)	<0.0001	<0.0001	
ever	ref	1.371 (0.631, 2.978)	0425	1.199 (0.565, 2.547)	0.637	2.034 (0.988, 4.184)	0.054	0.019	
Smoke status									
current	ref	1.777 (1.269, 2.487)	0.001	2.165 (1.538, 3.046)	<0.0001	3.208 (2.271, 4.532)	<0.0001	<0.0001	0.029
never	ref	1.031 (0.814, 1.306)	0.801	1.280 (1.023, 1.602)	0.031	1.570 (1.259, 1.958)	<0.0001	<0.0001	
ever	ref	1.189 (0.558, 2.535)	0.654	1.995 (0.976, 4.079)	0.058	3.009 (1.505, 6.015)	0.002	0.002	
BMI									
<24	ref	1.439 (1.108, 1.868)	0.006	1.635 (1.259, 2.124)	0.0002	2.597 (2.013, 3.351)	<0.0001	<0.0001	0.015
24-28	ref	0.991 (0.723, 1.358)	0.956	1.414 (1.059, 1.887)	0.0191	1.456 (1.080, 1.965)	0.014	0.004	
⩾28	ref	1.225 (0.725, 2.070)	0.448	1.560 (0.931, 2.612)	0.091	1.988 (1.203, 3.285)	0.007	0.002	
Hypertension									
no	ref	1.224 (0.975, 1.538)	0.081	1.516 (1.211, 1.899)	0.0003	2.205 (1.773, 2.743)	<0.0001	<0.0001	0.018
yes	ref	1.175 (0.849, 1.626)	0.329	1.495 (1.101, 2.031)	0.010	1.680 (1.233, 2.291)	0.001	0.0004	
Diabetes									
no	ref	1.324 (1.086, 1.616)	0.006	1.639 (1.348, 1.992)	<0.0001	2.113 (1.738, 2.569)	<0.0001	<0.0001	0.240
yes	ref	0.782 (0.461, 1.328)	0.362	1.055 (0.658, 1.692)	0.824	1.614 (1.028, 2.533)	0.037	0.0004	
Dyslipidemia									
no	ref	1.162 (0.929, 1.454)	0.188	1.435 (1.156, 1.782)	0.001	1.981 (1.595, 2.459)	<0.0001	<0.0001	0.646
yes	ref	1.418 (1.006, 1.999)	0.046	1.833 (1.311, 2.563)	0.0003	2.249 (1.622, 3.120)	<0.0001	<0.0001	
CKM									
0	ref	1.010 (0.221, 4.620)	0.990	4.436 (1.309, 15.040)	0.017	7.117 (2.108, 24.037)	0.002	0.0002	0.094
1	ref	1.017 (0.568, 1.820)	0.956	0.944 (0.534, 1.668)	0.842	1.144 (0.642, 2.039)	0.647	0.640	
2	ref	1.174 (0.846, 1.630)	0.337	1.257 (0.909, 1.738)	0.167	1.689 (1.235, 2.311)	0.001	0.0003	
3	ref	1.322 (1.027, 1.701)	0.030	1.808 (1.416, 2.307)	<0.0001	2.350 (1.840, 3.000)	<0.0001	<0.0001	

Table S17. Association between TyGFI and all-cause mortality stratified by sex, age, drink status, smoke status, BMI, hypertension, dyslipidemia, diabetes and CKM stages

character	Q1	Q2	p	Q3	p	Q4	p	p for trend	p for interaction
Sex									
male	ref	1.162 (0.474, 2.849)	0.744	2.054 (0.888, 4.747)	0.092	2.309 (1.001, 5.329)	0.050	0.020	0.569
female	ref	2.913 (0.618, 13.743)	0.177	2.583 (0.561, 11.892)	0.223	3.766 (0.875, 16.216)	0.075	0.081	
Age									
45-60	ref	1.692 (0.401, 7.149)	0.474	3.076 (0.809, 11.706)	0.099	4.577 (1.214, 17.250)	0.025	0.009	0.829
>60	ref	1.472 (0.605, 3.578)	0.394	0.675 (0.709, 3.958)	0.239	2.515 (1.098, 5.760)	0.029	0.009	
Drink status									
current	ref	0.892 (0.298, 2.675)	0.839	0.924 (0.306, 2.786)	0.888	1.649 (0.581, 4.675)	0.347	0.186	0.171
never	ref	2.714 (0.875, 8.416)	0.084	2.612 (0.856, 7.966)	0.092	3.949 (1.348, 11.570)	0.012	0.013	
ever	ref	-	-	-	-	-	-	-	
Smoke status									
current	ref	1.639 (0.560, 4.798)	0.368	2.278 (0.815, 6.366)	0.116	2.595 (0.920, 7.318)	0.071	0.067	0.949
never	ref	1.652 (0.505, 5.406)	0.406	1.585 (0.497, 5.056)	0.436	2.348 (0.795, 6.938)	0.122	0.095	
ever	ref	0.734 (0.057, 9.420)	0.812	2.271 (0.242, 21.346)	0.473	2.548 (0.278, 23.350)	0.408	0.155	
BMI									
<24	ref	1.444 (0.541, 3.849)	0.463	2.404 (0.965, 5.985)	0.060	3.325 (1.370, 8.068)	0.008	0.001	0.306
24-28	ref	1.530 (0.391, 5.988)	0.542	0.708 (0.173, 2.894)	0.631	1.398 (0.413, 4.730)	0.590	0.651	
⩾28	ref	-	-	-	-	-	-	-	
Hypertension									
no	ref	1.483 (0.551, 3.991)	0.436	2.009 (0.776, 5.200)	0.151	3.382 (1.364, 8.386)	0.009	0.001	0.288
yes	ref	1.426 (0.427, 4.757)	0.564	1.700 (0.546, 5.296)	0.360	1.632 (0.525, 5.078)	0.398	0.526	
Diabetes									
no	ref	1.342 (0.591, 3.044)	0.482	1.876 (0.861, 4.086)	0.113	2.395 (1.118, 5.131)	0.025	0.009	0.821
yes	ref	2.651 (0.293, 24.007)	0.386	2.242 (0.252, 19.954)	0.469	4.472 (0.567, 35.270)	0.155	0.076	
Dyslipidemia									
no	ref	2.279 (0.825, 6.295)	0.112	3.257 (1.237, 8.577)	0.017	4.524 (1.750, 11.695)	0.002	0.0003	0.048
yes	ref	0.756 (0.220, 2.598)	0.657	0.381 (0.100, 1.455)	0.158	0.758 (0.239, 2.409)	0.639	0.853	
CKM									
0	ref	-	-	-	-	-	-	-	0.084
1	ref	-	-	-	-	-	-	-	
2	ref	0.313 (0.047, 2.081)	0.229	0.096 (0.008, 1.155)	0.065	0.267 (0.045, 1.568)	0.143	0.375	
3	ref	1.716 (0.710, 4.148)	0.230	2.632 (1.143, 6.060)	0.023	3.362 (1.477, 7.650)	0.004	0.001	

Table S18. Multivariate cox regression for the correlation between TyGFI and CVD risk: complete-case analysis

TyGFI	Crude model		Model 1		Model 2	
	**HR (95% CI)**	***P***	**HR (95% CI)**	***P***	**HR (95% CI)**	***P***
CVD incidence						
Continuous	1.35 (1.28, 1.42)	< 0.0001	1.29 (1.22, 1.36)	< 0.0001	1.26 (1.19, 1.34)	< 0.0001
Categories						
Q1	Ref					
Q2	1.32 (1.10, 1.59)	0.003	1.27 (1.06, 1.53)	0.009	1.24 (1.03, 1.49)	0.024
Q3	1.67 (1.40, 1.99)	< 0.0001	1.58 (1.32, 1.88)	< 0.0001	1.52 (1.27, 1.81)	< 0.0001
Q4	2.47 (2.10, 2.92)	< 0.0001	2.18 (1.83, 2.60)	< 0.0001	2.05 (1.72, 2.45)	< 0.0001
P for trend		< 0.0001		< 0.0001		< 0.0001

Crude model: unadjusted for covariates; Model 1: age, gender, smoke, drink, sleep, WC, BMI, weight; Model 2: age, gender, smoke, drink, sleep, WC, BMI, weight, LDL-C, HBA1C, TC, HDL-C, eGFR, hypertension, diabetes, dyslipidemia.

Table S19. Multivariate cox regression for the correlation between TyGFI and all-cause mortality: complete-case analysis

TyGFI	Crude model		Model 1		Model 2	
	**HR (95% CI)**	***P***	**HR (95% CI)**	***P***	**HR (95% CI)**	***P***
All-cause mortality						
Continuous	1.67 (1.46, 1.91)	< 0.0001	1.40 (1.20, 1.63)	< 0.0001	1.41 (1.21, 1.64)	< 0.0001
Categories						
Q1	Ref					
Q2	2.19 (1.10, 4,48)	0.03	1.62 (0.79, 3.31)	0.190	1.45 (0.71, 2.99)	0.309
Q3	3.21 (1.63 ,6.31)	< 0.001	2.11 (1.07, 4.19)	0.032	1.77 (0.89, 3.54)	0.105
Q4	5.17 (2.71, 9.86)	< 0.0001	2.70 (1.39, 5.26)	0.003	2.49 (1.28, 4.86)	0.007
P for trend		< 0.0001		< 0.0001		< 0.0001

Crude model: unadjusted for covariates; Model 1: age, gender, marital, smoke, drink, sleep, height, weight; Model 2: age, gender, marital, smoke, drink, sleep, height, weight, CRP, TC, eGFR, cancer, hypertension, diabetes.

Table S20. Multivariate logistic regression for the correlation between TyGFI and CVD risk

TyGFI	Crude model		Model 1		Model 2	
	**OR (95% CI)**	***P***	**OR (95% CI)**	***P***	**OR (95% CI)**	***P***
CVD incidence						
Continuous	1.45 (1.37, 1.55)	< 0.0001	1.06 (1.05, 1.07)	< 0.0001	1.05 (1.04, 1.07)	< 0.0001
Categories						
Q1	Ref					
Q2	1.43 (1.17, 1.74)	< 0.001	1.04 (1.01, 1.07)	0.007	1.03 (1.01, 1.06)	0.022
Q3	1.82 (1.51, 2.21)	< 0.0001	1.08 (1.05, 1.11)	< 0.0001	1.07 (1.04, 1.10)	< 0.0001
Q4	2.90 (2.42, 3.49)	< 0.0001	1.16 (1.12, 1.19)	< 0.0001	1.14 (1.10, 1.18)	< 0.0001
P for trend		< 0.0001		< 0.0001		< 0.0001

Crude model: unadjusted for covariates; Model 1: age, gender, smoke, drink, sleep, WC, BMI, weight; Model 2: age, gender, smoke, drink, sleep, WC, BMI, weight, LDL-C, HBA1C, TC, HDL-C, eGFR, hypertension, diabetes, dyslipidemia.

Table S21. Multivariate cox regression for the correlation between TyGFI (additive model) and CVD incidence.

TyGFI	Crude model		Model 1		Model 2	
	**HR (95% CI)**	***P***	**HR (95% CI)**	***P***	**HR (95% CI)**	***P***
All-cause mortality						
Continuous	1.69 (1.47, 1.94)	< 0.0001	1.01 (1.00, 1.01)	< 0.0001	1.01 (1.00, 1.01)	< 0.001
Categories						
Q1	Ref					
Q2	2.20 (1.10, 4.69)	0.031	1.00(0.99, 1.01)	0.557	1.00(0.99, 1.01)	0.707
Q3	3.23 (1.69, 6.70)	< 0.001	1.01(1.00, 1.02)	0.178	1.00(1.00, 1.01)	0.354
Q4	5.24 (2.85, 10.59)	< 0.0001	1.01(1.00, 1.02)	0.006	1.01(1.00, 1.02)	0.032
P for trend		< 0.0001		< 0.0001		0.031

Crude model: unadjusted for covariates; Model 1: age, gender, marital, smoke, drink, sleep, height, weight; Model 2: age, gender, marital, smoke, drink, sleep, height, weight, CRP, TC, eGFR, cancer, hypertension, diabetes.

Table S22. Post-Hoc Power Calculations to assess the efficacy of observed associations

Outcome	Sample Size	Event Count	Q4 vs Q1 TyGFI HR	α	Power
CVD	6207	4594	2.02	0.05	≈ 100%
Dead	6386	6269	2.67	0.05	≈ 100%

Table S23. Multivariate cox regression for the correlation between TyGFI (additive model) and CVD incidence.

**TyGFI**	**Crude model**		**Model 1**		**Model 2**	
	**95% CI**	***P***	**95% CI**	***P***	**95% CI**	***P***
CVD						
Continuous	1.33 (1.23,1.43)	< 0.0001	1.22 (1.12,1.32)	< 0.0001	1.09 (0.95,1.24)	0.218
Categories						
Q1	Ref					
Q2	1.35 (1.13,1.60)	< 0.001	1.23 (1.03,1.47)	0.019	1.19 (0.98,1.45)	0.078
Q3	1.68 (1.42,1.99)	< 0.0001	1.43 (1.20,1.69)	< 0.0001	1.34 (1.06,1.70)	0.015
Q4	1.76 (1.49,2.08)	< 0.0001	1.40 (1.17,1.66)	< 0.0001	1.08 (0.76,1.53)	0.660
P for trend		< 0.0001		< 0.0001		0.031

Crude model: unadjusted for covariates; Model 1: age, gender, smoke, drink, sleep, WC, BMI, weight; Model 2: age, gender, smoke, drink, sleep, WC, BMI, weight, LDL-C, HBA1C, TC, HDL-C, eGFR, hypertension, diabetes, dyslipidemia.

Table S24. Multivariate cox regression for the correlation between TyGFI (additive model) and all-cause mortality.

**TyGFI**	**Crude model**		**Model 1**		**Model 2**	
	**95% CI**	***P***	**95% CI**	***P***	**95% CI**	***P***
CVD						
Continuous	0.95 (0.73,1.23)	0.685	1.08 (0.80,1.45)	0.631	0.95 (0.67,1.35)	0.768
Categories						
Q1	Ref					
Q2	0.91(0.56,1.48)	0.711	0.92(0.55,1.52)	0.737	0.93(0.55,1.56)	0.784
Q3	0.88(0.54,1.44)	0.616	0.82(0.48,1.38)	0.453	0.85(0.49,1.46)	0.546
Q4	0.93(0.58,1.49)	0.709	0.16(0.69,1.97)	0.571	0.98(0.53,1.81)	0.941
P for trend		0.292		0.875		0.489

Crude model: unadjusted for covariates; Model 1: age, gender, marital, smoke, drink, sleep, height, weight; Model 2: age, gender, marital, smoke, drink, sleep, height, weight, CRP, TC, eGFR, cancer, hypertension, diabetes.

Table S25. Multivariate cox regression for the correlation between TyGFI and CVD incidence during a four-year follow-up

TyGFI	Crude model		Model 1		Model 2	
	**95% CI**	***P***	**95% CI**	***P***	**95% CI**	***P***
CVD						
Continuous	1.35 (1.24, 1.46)	< 0.0001	1.34 (1.23, 1.46)	< 0.0001	1.26 (1.15, 1.38)	< 0.0001
Categories						
Q1	Ref					
Q2	2.14 (1.53, 2.99)	< 0.0001	2.04 (1.46, 2.87)	< 0.0001	1.92 (1.36, 2.72)	0.0002
Q3	2.33 (1.67, 3.24)	< 0.0001	2.20 (1.57, 3.08)	< 0.0001	2.03 (1.41, 2.95)	0.0002
Q4	3.43 (2.50, 4.70)	< 0.0001	2.97 (2.13, 4.14)	< 0.0001	2.51 (1.53, 4.13)	0.0003
P for trend		< 0.0001		< 0.0001		< 0.0001

Crude model: unadjusted for covariates; Model 1: age, gender, smoke, drink, sleep, WC, BMI, weight; Model 2: age, gender, smoke, drink, sleep, WC, BMI, weight, LDL-C, HBA1C, TC, HDL-C, eGFR, hypertension, diabetes, dyslipidemia.

Table S26. Multivariate cox regression for the correlation between TyGFI and all-cause mortality during a four-year follow-up

TyGFI	Crude model		Model 1		Model 2	
	**HR (95% CI)**	***P***	**HR (95% CI)**	***P***	**HR (95% CI)**	***P***
All-cause mortality						
Continuous	1.74 (1.49, 2.03)	< 0.0001	1.41 (1.17, 1.69)	0.0002	1.41 (1.17, 1.70)	0.0003
Categories						
Q1	Ref					
Q2	2.26 (0.98, 5.20)	0.055	1.89 (0.78, 4.54)	0.157	1.55 (0.64, 3.78)	0.333
Q3	2.89 (1.29, 6.46)	0.010	2.07 (0.88, 4.90)	0.096	1.64 (0.68, 3.91)	0.269
Q4	5.31 (2.49, 11.30)	< 0.0001	2.91 (1.27, 6.66)	0.011	2.53 (1.10, 5.81)	0.029
P for trend		< 0.0001		< 0.0001		< 0.0001

Crude model: unadjusted for covariates; Model 1: age, gender, marital, smoke, drink, sleep, height, weight; Model 2: age, gender, marital, smoke, drink, sleep, height, weight, CRP, TC, eGFR, cancer, hypertension, diabetes.
